# Risk factors associated with sexually transmitted infections in Nigeria: a systematic review

**DOI:** 10.1186/s12889-026-26918-z

**Published:** 2026-03-09

**Authors:** Abel Onolunosen Abhadionmhen, Zakari Isiaka Osheku, Edobor Peter Kenneth Imarenezor

**Affiliations:** 1 Department of Microbiology, Federal University Wukari, Wukari, Taraba State Nigeria; 2 Primary Health Care Initiative Africa, Kaduna, Kaduna State Nigeria; 3 Department of Microbiology, Federal University Otuoke, Otuoke, Bayelsa State Nigeria

**Keywords:** Nigeria, Sexually transmitted infections, Risk factors, Sexual health, Contraception

## Abstract

**Background:**

Sexually transmitted infections (STIs) pose a major public health challenge globally, with Nigeria experiencing a significant burden. Despite on-going sexual health interventions, the determinants of STI risk across diverse Nigerian populations remain insufficiently understood. This systematic review synthesizes current evidence to inform policy and intervention strategies.

**Methods:**

A comprehensive search of PubMed, Web of Science, EMBASE, and AJOL used Boolean operators and STI-related keywords. Eligible peer-reviewed studies, conducted in Nigeria between 2014 and 2023, examined STI risk factors and used qualitative, quantitative, or mixed-methods designs. Studies were screened using predefined criteria, appraised with JBI tools, and synthesized through narrative and thematic approaches.

**Results:**

Twenty-three studies met the inclusion criteria and highlighted multiple influences on STI risk in Nigeria. Socioeconomic conditions, including education and employment, shaped access to contraceptives and sexual health services. Cultural expectations and gender norms affected attitudes and behaviours related to protection. Structural challenges such as condom unavailability and limited sexual health information were more pronounced in rural settings. Interpersonal dynamics, including partner communication and decision-making capacity, influenced protective behaviour, while substance use and low risk perception increased STI vulnerability.

**Conclusion:**

STI risk in Nigeria is shaped by interconnected socioeconomic, cultural, structural, interpersonal, and behavioural factors. Strengthening sexual health education, addressing gender disparities, improving contraceptive access, and integrating mental health support are critical for reducing STI burden. The review is registered with PROSPERO (CRD42024551254).

**Supplementary Information:**

The online version contains supplementary material available at 10.1186/s12889-026-26918-z.

## Background

 Sexually transmitted infections (STIs) are infections that are primarily spread through sexual contact, including vaginal, anal, and oral sex [1–3]. These infections can be caused by bacteria, viruses, or parasites and can affect people of all ages, genders, and sexual orientations [4, 5]. STIs are a significant global health burden, with more than one million new cases acquired daily among individuals aged 15–49 years, with a significant portion being asymptomatic [2]. In 2020 alone, there were an estimated 374 million incidences of STIs, with one in four involving chlamydia, gonorrhea, syphilis, and trichomoniasis [4]. Specifically, in 2022, approximately 8 million adults aged 15 to 49 years were infected with syphilis [2]. The burden of STIs is disproportionally greater in low- and middle-income countries, with sub-Saharan Africa contributing 93 million new cases [2, 6]. Young people face a disproportionate risk due to behaviors and limited access to healthcare [7].

In Nigeria, the National Agency for the Control of AIDS (NACA) reported a 3.0% HIV prevalence among adults aged 15–24 [8]. Gonorrhea (3.5%) and chlamydia (5%) prevalence rates continue to increase, with the highest growth observed in urban areas. According to the available data, the prevalence of trichomoniasis in Nigeria is 25.9% [9]. Among young women aged 15–24 years in Akwa Ibom State, Nigeria, the prevalence rate is notably lower, at 6.8% [10]. Genital herpes, while not as extensively studied, is recognized as a common STI in Nigeria, further exacerbating the burden faced by young Nigerians [11].

High STI rates have significant public health implications, leading to immediate and long-term health issues such as pelvic inflammatory disease, infertility, and chronic pelvic pain [12]. Untreated STIs can also cause severe neonatal infections and congenital anomalies, increasing infant morbidity and mortality [13]. Economically, STIs strain healthcare resources, diverting them from other priorities and hindering system efficiency [14]. Additionally, untreated STIs contribute to stigma, mental health issues, and barriers to education and employment, perpetuating poverty and impeding social and economic development, especially among individuals in rural communities [15].

Risky sexual behaviors such as early sexual debut, multiple sexual partners, inconsistent condom use, transactional sex and substance abuse are significant contributors to high STI rates [16]. Similarly, socioeconomic factors such as poverty, limited education access, and a lack of economic opportunities, such as peer pressure, exacerbate these risky behaviors, which are prevalent among 63% of Nigerian adolescents [16, 17]. Furthermore, cultural norms and stigmatization hinder open discussions and access to sexual health services [18]. Structural disparities, including inadequate healthcare infrastructure in rural areas, a lack of concerted and comprehensive services, and insufficient sexual health education, create barriers to prevention and treatment [19].

Studying the sexual health of individuals across various demographics in Nigeria is crucial due to their vulnerability to STIs [7, 20]. A thorough understanding of their challenges allows for the creation of tailored public health policies aimed at achieving better outcomes [21]. A preliminary literature search revealed limited data on systematic reviews or meta-analyses summarizing the determinants of sexual health risk factors in Nigeria. However, the individual research landscape was fragmented, hindering broad conclusions and unified strategies. This gap impedes the understanding of all determinants influencing STI risk in Nigerian. Hence, this systematic review aimed to identify and analyze the determinants of STI risk factors in Nigerian. Additionally, this review contributes to public health knowledge and policy by synthesizing current evidence, highlighting key risk factors, and identifying research gaps. Our findings will support the development of stronger public health policies and programs, leading to improved sexual health outcomes for individuals in Nigeria. Ultimately, this study will identify knowledge gaps, guide future research and emphasize the need for targeted interventions.

## Methods

### Study design

The protocol for this systematic review was registered with the International Prospective Register of Systematic Reviews (PROSPERO) under the registration number CRD42024551254. We clearly defined and presented the protocol for this review following the Preferred Reporting Items for Systematic Reviews and Meta-Analyses (PRISMA) guidelines [22].

### Search strategy

We developed a systematic search strategy to comprehensively identify the literature for this review. The search terms were carefully selected to capture a wide range of relevant studies. We searched several academic databases on 17th May, 2024, including PubMed, Web of Science, EMBASE, and AJOL. Boolean operators (AND, OR) and parentheses were used to structure the search effectively, allowing for the inclusion of various combinations of keywords. The search query was formulated to include all relevant terms and ensure comprehensive coverage of the literature. The final search string was as follows: (adolescent OR youth OR “young adult*” OR adult* OR student* OR “sexually active” OR “young people) AND (“STI risk factors” OR “risky sexual behavior” OR “sexual health” OR “sexually transmitted infections” OR sexual behavior OR sexual activity OR sexual practices OR sexual intercourse OR sexual relationships OR sexual risk-taking OR sexual attitudes) AND (“condom use” OR “contraceptive” OR condom usage OR safe sex practices OR birth control OR family planning OR contraception methods OR preventive measures OR reproductive health OR unprotected sex OR sexual protection”) AND Nigeria. (See supplementary material)

### The eligibility criteria

We included peer-reviewed studies conducted in Nigeria that examined risk factors for STI involving adolescents, young adults, or adult populations as defined by the primary studies. Eligible articles were published in English between 2014 and 2023 and used qualitative, quantitative, or mixed-methods research designs. Studies that did not meet the inclusion criteria, including non-peer-reviewed articles, systematic reviews, letters, and those not specifically addressing the themes of STI risk factors, were excluded.

### Selection and data extraction

The search results were imported into an EndNote library, where duplicate entries were methodically eliminated to ensure that the library comprised only unique references, enhancing research organization and accuracy. AOA independently managed the initial assessment of titles and abstracts, with ZIO conducting a separate, independent check. ZIO then independently led the retrieval and evaluation of the full-text articles using the predefined criteria, with AOA performing an independent verification. Discrepancies arising at either the title/abstract stage or the full-text stage most commonly related to borderline eligibility or unclear reporting of key study characteristics were resolved through discussion with a third reviewer (EPKI), who provided final adjudication where needed. Subsequently, the AOA and ZIO thoroughly analyzed selected studies to identify key themes regarding STI risk factors and risky sexual behavior. EPKI supervised the synthesis and comparison of these themes across studies to identify patterns and relationships. ZIO created a comprehensive table for each included study, outlining details such as author names, publication year, participant demographics, study design, study quality, key findings related to STI risk factors, risky sexual behaviors, and any recommendations for public health interventions or policy changes.

### Critical appraisal of studies

We conducted a rigorous appraisal of the selected literature using the Joanna Briggs Institute (JBI) tool [23]. AOA and ZIO assessed the quality of the studies, focusing on sample size, study design, data collection methods, and analysis techniques. The JBI tool for cross-sectional studies encompassed 8 items, while qualitative studies involved 10–12 items, addressing study design, data collection, and interpretation of findings. Utilizing the original JBI tools without alterations, a binary system of “yes” or “no” indicated criterion presence or absence, “unclear” indicated insufficiently detailed criteria, and “not applicable” indicated irrelevant criteria. Despite the tool lacking predefined thresholds, we classified studies as low, moderate, or high quality based on specific criteria. High methodological quality for cross-sectional studies had scores between 7 and 8, moderate quality had scores between 4 and 6, and low quality had scores below 4. For qualitative research, high-quality studies had scores ranging from 9 to 10, medium-quality studies had scores ranging from 5 to 8, and low-quality studies had scores below 5. Irrespective of the scores, no literatures were excluded based on quality. The evaluations involved thorough review and discussion with EPKI to ensure consistency and accuracy.

### Data analysis

Our study employed qualitative narrative approach supported by thematic and line-of-argument synthesis to identify patterns across studies. AOA led the initial scrutiny by meticulously extracting significant codes from each study, focusing on specific aspects of STI risk factors. Subsequently, ZIO consolidated similar codes into distinct subthemes and further refined them into broader categories through rigorous thematic analysis. Discrepancies were unanimously resolved with input from the third reviewer, EPKI, who ensured methodological integrity by overseeing thematic refinement and triangulation. This process guaranteed the accuracy and relevance of the themes aligning with the procedural guidelines and techniques of grounded theory [24]. A fishbone diagram visually mapped the identified themes, while a thematic matrix ensured consistency and comprehensive coverage. A line-of-argument synthesis was constructed to explore the interrelationships between themes, drawing on pertinent public health theories related to sexual health. The final step involved synthesizing these themes to extract meaningful insights and facilitate further discussion. The synthesis was informed by public health theories, including the Social Determinants of Health (SDH), the Ecological Model of Health Promotion (EMHP), the Health Belief Model (HBM), the Health Promotion Model (HPM), and the Parental Involvement Theory (PIT), which served as interpretive lenses for organizing and interpreting determinants reported across studies.

## Results

### Attributes of the incorporated literature

**Fig. 1:**
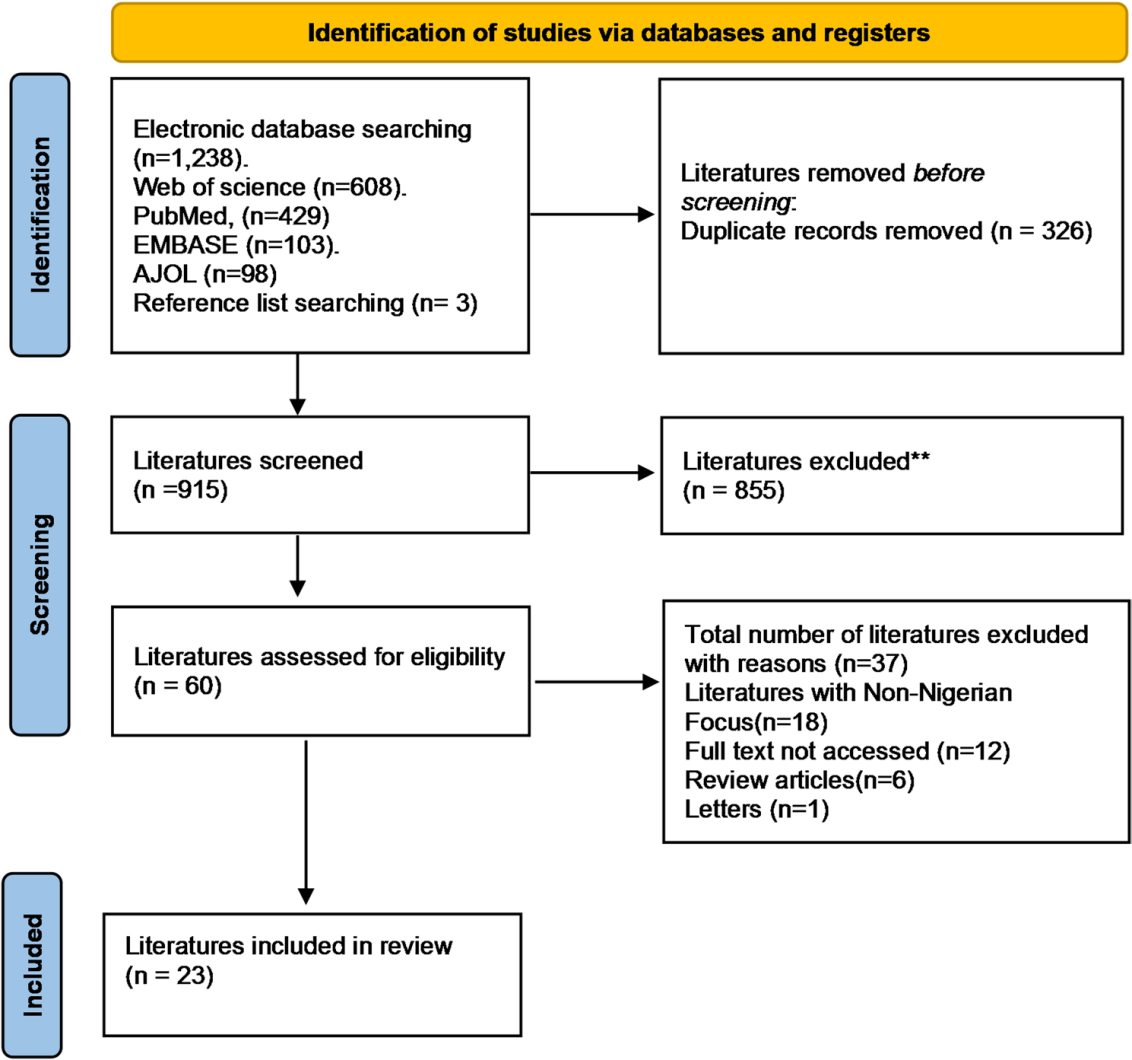
Literature selection flow diagram

A thorough literature search was performed across various electronic databases, as illustrated in the PRISMA flow chart in Fig. 1, resulting in the identification of 1,238 articles. Three additional articles were identified through a reference list search, resulting in a total of 1,241 articles. During the screening stage, 326 duplicate articles were excluded, leaving 915 articles for further evaluation. A detailed eligibility assessment was conducted on these 915 articles, leading to the exclusion of 855 articles that did not meet the predefined inclusion criteria. Subsequently, the full texts of the remaining 60 articles were reviewed. This process resulted in the exclusion of 37 articles for the following reasons: 18 articles had a non-Nigerian focus, 12 articles’ full texts were not accessible, 6 articles were review articles, and 1 article was a letter. Ultimately, 23 articles met all the criteria and were included in the review. A summary of the characteristics and key findings of the included studies is presented in Table 1.

**Table 1:** Summary of the characteristics and key findings of the included literatures

SN	Author	Aim of research	Population	Method	Findings (STI Risk Factors)	Conclusion
1	Akamike et al., 2020 [27]	To assess the factors influencing family planning uptake among married women in rural communities	The study population included women of reproductive age (15–49 years)	Cross sectional	Family planning use was low overall, with natural methods preferred. Among modern methods, condom use remained limited, indicating significant gaps in access to and acceptance of protective contraceptive options.	Perception and use of condom and other family planning methods is poor in communities studied. Predisposing individuals to STI
2	Ajayi et al., 2019 [28]	To evaluate the consistency of condom, use and its determinant among young adults in two Nigerian universities.	The sample consisted of 498 male and female students who engaged in sex in the past year.	Cross sectional	Condom use consistency was generally low. Greater self-efficacy, open discussions about HIV, awareness of a partner’s HIV status, and stable relationships supported more consistent use, while trust, limited availability, dislike, and perceived reduction in pleasure contributed to inconsistent use.	Low consistent condom use among participants was observed. Campus condom availability and STI discussions will improve usage.
3	Ajayi and Okeke 2019 [29]	To examine the influence of family support and gender differences in protective sexual behaviors among adolescents and young adults in Nigerian Universities.	The study included 599 university students aged 15 to 24 from two Nigerian Universities.	Cross sectional	Most adolescents reported strong family support, yet notable proportions engaged in substance use and risky sexual behaviors. While some abstained from sex, a significant minority exhibited high-risk practices such as inconsistent condom use and lack of sexual fidelity.	Most Nigerian university students practice protective sexual behaviors with family support, yet about one-fifth engage in high-risk behaviors, necessitating targeted Interventions.
4	Blackstone and Iwelunmor 2017 [30]	To investigate determinants of contraceptive use in Nigerian couples.	The study included a total of 38,948 women, 17,359 men, and 8658 couples who are within the ages of 15–49	Cross sectional	Contraceptive use among couples was generally low. Traditional beliefs and gendered misconceptions were widespread, with many men viewing contraception as solely a woman’s responsibility and associating its use with promiscuity, highlighting persistent cultural barriers to modern contraceptive uptake.	Male partners play a significant role in decisions regarding women’s contraceptive use and emphasize the importance of their involvement in family planning initiatives in Nigeria.
5	Mbachu et al., 2021 [25]	To explore beliefs and misconceptions about condoms and other contraceptives among adolescents	The study involved 124 in- and out-of-school adolescents aged 13–18 years	A qualitative study using twelve focus group discussions (FGD	Adolescents knew about contraception and condom use but held misconceptions, such as preventing pregnancy with hard drugs and laxatives, and believed condoms were reusable and reduced pleasure, leading some to prefer withdrawal.	Despite knowledge of contraception, misconceptions persist; increasing pregnancy and STI risks, highlighting the need for educational interventions to correct these views and promote safe sex practices.
6	Adogu, et al., Sexual health, 2023 [31]	To compare sexual health knowledge, attitudes, and risk perception among in-school and out-of-school female adolescents	A total of 783 adolescents were included in the study.	Cross sectional	In-school girls had greater parental support and better awareness of contraceptives and condoms, while out-of-school girls demonstrated poorer HIV-risk understanding. Risky sexual behaviors were more common among out-of-school adolescents, indicating the need for strengthened sexual-health education and risk-perception interventions.	The study determined that more misconceptions persist, highlighting the need for interventions to improve sexual health knowledge and risk perception.
7	Adeomi et al., 2014 [32]	To describe sexual risk behaviors among adolescents attending secondary schools in a Southwestern State in Nigeria.	A total of 815 in-school adolescents was included in the study.	Cross sectional	A notable proportion of adolescents were already sexually active, many beginning at an early age. Risky practices such as recent sexual activity, transactional sex, and low condom use were common, and several reported symptoms suggestive of STIs.	There is a high level of sexual risk behavior among these adolescents, with many initiating sexual activities at a young age. Public health interventions should target early adolescents to prevent HIV/AIDS and other STIs.
8	Folayan et al., 2022 [26]	To understand the determinants and barriers to addressing adolescent sexual and reproductive health (SRH) and HIV prevention needs in Nigeria.	A total of 49 involving adolescents, healthcare workers, civil society members, and parents of HIV positive adolescents	Qualitative focus group discussions FGD	Identified factors influencing sexual risk behaviors, including peer pressure and misconceptions about contraceptives; highlighted shortcomings in health systems’ ability to address adolescents’ nee	More robust research is necessary to grasp the interplay between adolescent risk behavior, HIV risk perception, parental involvement in mitigating HIV risk, and the community and government’s roles in HIV prevention and treatment for Nigerian adolescents.
9	Omisore et al., 2022 [33]	To identify factors associated with risky sexual behavior (RSB) among undergraduates in Osun state.	A toral of 550 respondents from two universities were involved in the study	Cross sectional	Risky sexual behavior was widespread among respondents. Males and youths who used alcohol or drugs, consumed pornography, spent significant time online, or had strained relationships with their mothers were more likely to engage in risky sexual practices.	RSB is common among undergraduates, with males more involved. Stakeholders should use behavioral change strategies to reduce youth engagement in RSB.
10	Oharume 2020 [34]	To examine the knowledge, sexual behaviors, and risk perception of STIs among students of the polytechnic, Ibadan, Nigeria.	A total of 401 students participated in the survey.	Cross sectional	Most students had poor STI knowledge and many were sexually active, with a considerable proportion engaging in high-risk practices such as multiple partnerships and inconsistent condom use. Very few perceived themselves as being at risk of contracting STIs.	These results emphasize the need to provide students with more information about STIs with the aim of positively influencing their self-perceived risk
11	Adejumo et al., 2022 [35]	To determine the prevalence of risky sexual behavior and its associated factors among clients accessing HIV counseling and testing services	A retrospective review of 4273 client records accessing HIV counseling and testing services	Cross sectional	Risky sexual behavior was common among clients, especially males and those living with HIV. Single individuals were more likely to engage in unprotected sex with casual or multiple partners. Risky practices tended to decline with increasing age.	Age, marital status, and HIV status were identified as associated factors of risky sexual behavior among clients accessing HIV counseling and testing services.
12	Eyam et al., 2022 [36]	To determine the prevalence and determinants of risky sexual behavior among adolescents in coeducational public secondary schools	The study included a total of 768 adolescents aged 10–19 years in coeducational public secondary schools without sexuality education programs in Cross River State.	Cross sectional	Risky sexual behavior was common among adolescents and more frequent among boys. Older adolescents, those from polygamous homes, students nearing graduation, and youths with limited parental monitoring or lower maternal education were especially vulnerable. Age and family structure were major determinants of risk.	High prevalence of risky sexual behavior among adolescents. Age, family type, parental monitoring, and socioeconomic status are significant factors.
13	Osuala et al., 2021 [37]	Determine the prevalence of risky sexual behavior among students in tertiary institutions in Rivers State, Nigeria	A total of 280 participants who are year one and two Medical and Nursing undergraduate students	Cross sectional	A substantial number of undergraduates were already sexually active, many beginning at an early age. Condom use was inconsistent, and some students reported condom breakage and the use of drug enhancers during sex, indicating widespread engagement in risky sexual practices.	Undergraduates engage in risky sexual behaviors, necessitating improved public health strategies on health education and reproductive services.
14	Okunlola et al., 2020 [38]	To identify sociodemographic, economic, and psychological factors linked to risky sexual behavior among sexually active Nigerian youths	A total of 7,909 sexually active youths in Nigeria	Cross sectional	Early sexual debut was common among youths, with many engaging in unprotected sex and multiple partnerships. Risky sexual behavior was strongly shaped by sociodemographic, economic, and psychological factors.	Sociodemographic, economic, and psychological factors predict Nigerian youths’ risky sexual behavior, varying by behavior type; interventions must address these factors to reduce risks.
15	Adedini et al., 2021 [39]	To assess changes in sexual and reproductive health (SRH) behaviors of unmarried young people aged 15–24 and associated factors over a ten-year period in Nigeria.	The study population included 3,991 men and 7,356 women, aged 15–24, sampled across the 2008, 2013, and 2018 NDHS	Cross sectional	Risky sexual behavior among unmarried young Nigerians increased over the ten-year period. However, factors such as better HIV knowledge, being male or older, urban residence, higher socioeconomic status, and higher education were associated with greater protection against risky practices.	Surging rates of risky sex among Nigerian youth demand interventions targeting the most vulnerable.
16	Akokuwebe et al., 2019 [40]	To assess knowledge, attitudes, and sexual coercion among rural in-school adolescents. Evaluate their confidence in adopting safer sex methods.	A total of 279 in-school rural adolescents	Cross sectional	Many adolescents began sexual activity unintentionally and engaged with multiple partners. Most viewed abstinence as a safe method, and many learned about sex from peers or social media. Sexual behavior was shaped by gender, age, education level, family structure, place of residence, and parents’ occupation.	Unsafe practices were predicted by gender, age, education, family, residence, and parents’ occupation. Urgent need for targeted sexual health education.
17	Osadolor et al., 2022 [41]	To assess the effect of exposure to sex education on adolescents’ sexual behavior	A total of 345 adolescents were involved in this study	Cross sectional	School was the primary source of sex education for most adolescents, followed by family and media. More frequent discussions about sexual matters were linked to better protective practices, including condom use.	The study advocates for expanded sexuality education, acknowledging its ability to positively impact adolescents’ sexual behavior
18	Alo et al., 2020 [42]	To investigate the factors influencing the use of modern contraceptive methods among sexually active women aged 15–49 in Nigeria	The study included 9126 sexually active women aged 15–49 across 295 clusters	Cross sectional	Use of modern contraceptives was generally low, with condoms being the most commonly used method. Modern contraceptive uptake was strongly influenced by women’s education, marital status, socioeconomic status, and awareness of family planning options.	The study stressed on the urgent need for comprehensive strategies aimed at addressing disparities in family planning utilization based on socioeconomic status
19	Chingle et al., 2017 [43]	To assess condom utilization and predictors of condom use among male respondents in Plateau State, Nigeria	The study involved 393 consenting males aged 15–49 years in Plateau State.	Cross sectional	Awareness of family planning was high, but actual condom use remained low. Condom use was shaped by several factors, including age, wealth, education, marital status, occupation, and religion. Men who were unmarried, of middle-class status, or had primary education were more likely to use condoms.	Male condom use in Plateau State is low, indicating a need for targeted interventions.
20	Ochonye et al., 2019 [44]	To assess the sexual risk profiles of female sex workers (FSW), men who have sex with men (MSM), and people who inject drugs (PWID)	The study population included 188 FSW, 145 MSM, and 155 PWID, totaling 488 individuals.	Cross sectional	Condom use was lowest among FSW and PWID, especially during anal sex. High levels of psychoactive drug use and needle sharing increased risk, while substance use and weak condom-negotiation skills further reduced protective behavior.	HIV prevention programs for MSM, FSW, and PWID should address inconsistent condom use by enhancing condom negotiation skills, as this risk behavior is common across all three groups.
21	Uchendu et al., 2019 [45]	To assess awareness and use of female condoms among street youths aged 15 to 24 in the sub-Saharan region.	A total of 964 youths were involved in this study.	Cross sectional	Most participants were sexually active, yet awareness and use of female condoms were very low. Awareness was influenced by age, education, sexual activity, and experiences of sexual violence.	Awareness of female condoms was a significant predictor of their use, highlighting the need for improved education on their effectiveness.
22	Odimegwu & Somefun, 2017 [46]	To examine the relationship between ethnicity and youth sexual reproductive health	The study included 6304 females and 1549 males aged 15–24 who are sexually active	Cross sectional	Females tended to begin sexual activity slightly earlier than males, with condom use far lower among females. Multiple partnerships were more common among males. Certain ethnic groups—specifically Hausa/Fulani females and Yoruba males—were at higher risk of early sexual activity.	The study highlights the necessity of tailored interventions to promote safe sexual practices among Nigerian youth, focusing on HIV and STI prevention.
23	Ajayi & Akpan, 2018 [47]	To investigate the rates of condom use and its determinants among parous women in three states in North Central and South Western Nigeria.	The study population consists of 1227 parous women in North Central and living in Ekiti State, and South Western Nigeria.	Cross sectional	Condom use among parous women was generally low but higher among those already using other contraceptives. Use was more common among women aged 26–35, urban residents, those without income, and those with higher education, reflecting a gradual increase in condom adoption.	The study highlights the increasing trend of condom use among parous women and identifies demographic and socioeconomic factors associated with condom use.

Two of the included literatures [25, 26] were qualitative study while the remaining twenty-two [27–47] used cross-sectional research methods. Notably, five of these cross-sectional studies [30, 38, 39, 42, 46] used secondary data. The sample sizes of the included studies ranged from 124 to 64,965, with a total of 115,401 participants, covering diverse populations and settings in Nigeria. Additionally, 49 focus groups were involved in the qualitative study discussions of [26].

The selected literature reflects a broad geographic distribution, covering multiple states across Nigeria’s six geopolitical zones. Specifically, studies [25, 27] were conducted in Ebonyi State, while [28–30] took place in Ilorin, Kwara State. Studies [31, 36] were carried out in Onitsha, Anambra State, and the Cross River State. Studies [32, 33] were conducted in Osun State, and [26, 40] in Lagos State, with [26] also including participants from Borno, Edo, Imo, and Kano States. Research in Oyo State was represented by [34, 45], while [47] included participants from Ondo and Ekiti States. In Rivers State, studies were conducted by [37, 41], and in Ogun State, they were conducted by [40]. Plateau State was the location for study [43], while [44] took place in Enugu, Nasarawa, Benue, and Akwa-Ibom States. Additionally [28, 29, 35, 44], were conducted in Nasarawa State. Despite the regional diversity, five articles drew data from nationally representative surveys, including the Nigeria Demographic and Health Surveys (NDHS) [30, 39, 46], the Nigeria Multiple Indicator Cluster Survey (MICS) [38], and linked household and Service Delivery Point (SDP) datasets [42]. Additionally, study [26] involved the analysis of transcripts from nationally conducted focus group discussions (FGDs).

### Critical appraisal of the included literature

Quality appraisal was conducted using the Joanna Briggs Institute (JBI) critical appraisal tools appropriate for each study design. Twelve cross-sectional studies [27–30, 33, 35, 36, 39, 42, 43, 46, 47] achieved high methodological quality, scoring 8 out of 8 on the JBI checklist. An additional five cross-sectional studies [31, 38, 40, 44, 45] also demonstrated high quality with scores of 7 out of 8. Three cross-sectional studies [32, 34, 41] were rated as moderate quality, scoring between 5 and 6 out of 8. The qualitative studies [25, 26] were appraised as moderate quality, each scoring 7 out of 10 on the JBI tool.

Across the included studies, common methodological limitations included reliance on self-reported sexual behaviors and inadequate control for potential confounders. Nevertheless, no study fell below the threshold for moderate quality, and all included evidence demonstrated acceptable methodological rigor. These quality assessment findings support the reliability of the narrative synthesis of risk factors associated with STI in Nigeria.

### Risk factors associated with STIs

#### Low and inconsistent condom use

Condom use rates vary significantly across studies. Studies [27, 28, 30, 31, 33, 42, 43, 45–47] reported rates of 48.1%, 21%, 38.6%, 29.5%, 32.7%, 9.2%, 15.5%, 13.8%, 4.3%, and 14.5%, respectively. Studies [28, 32, 34] have reported inconsistent condom use rates of 18%, 23.1%, and 26.7%, respectively. Furthermore, men who have sex with men (MSM) and people who inject drugs (PWID) were less likely than female sex workers (FSW) to use condoms consistently [44].

#### Risky sexual behavior

Trends in risky sexual behavior, including inconsistent condom use, multiple sex partners, and early sexual debut, were documented across fourteen studies. The rates of having multiple sexual partners varied among individuals, as reported by previous studies: 64% [40], 81% [46], 44% [37], 32% [38], 26% [34], and 19.7% [28]. Additionally, 14.2% of participants in [34] considered themselves at risk of contracting STIs, while 3.7% engaged exclusively with commercial sex workers, indicating an elevated risk of STI transmission within this subgroup [28]. The average age at sexual initiation among adolescents was 12.7 years [38], with approximately 66% experiencing their first intercourse before turning 18 [32] and 14.9% at the age of 15 [37].

Furthermore, a high proportion of psychoactive drug use was reported among MSM (23.5%) and FSW (23.4%), with recent injection drug use observed in 25.0% of FSW and 29.4% of MSM [44]. Additionally, 39.3% of PWID shared needles [44]. About 42% of adolescents aged 14–16 years were found to be more involved in risky sexual behavior [36]. Similarly, over 80% of unmarried young individuals reported engaging in risky sexual behaviors [39]. Alcohol consumption was reported by 65.8% of university students in one study [33] and 26.5% in another [29]. Additionally, heavy reliance on illicit drugs (16.5%) and tobacco use (12.9%) were identified as contributing factors to risky sexual behaviors among university students [29]. Finally, the use of sex-enhancer drugs [33] and pornography [37] also significantly influenced risky sexual behavior.

#### Poor awareness and knowledge of STI

Awareness and understanding of sexual health was identified to significantly influence the sexual behaviors of individuals. Peer influence and misconceptions about sexual health, such as the belief that hard drugs and laxatives could prevent pregnancy and that condoms were reusable, contributed to drug abuse, smoking, and early sexual debut [26, 43]. Individuals were found to primarily receive sexual education from various sources, with schools being the most common (54.6%), followed by family (21.6%) and other media platforms such as television (7.6%), books, or magazines (4.9%) [41]. Social media also played a significant role in providing sexual health education, with 33% [40] and 9.5% [41] of adolescents reporting it as a source of information. Additionally, some adolescents learn about sex education from their peers [41]. Higher socioeconomic status and education positively influence healthy sexual behaviors [39]. However, studies have shown that only 18.7% of polytechnic students have good knowledge of STIs [34]. Risky sexual behavior is prevalent among undergraduates in tertiary institutions [29, 33, 37]. Nonetheless, in-school girls demonstrated superior knowledge of sexual and reproductive health, including fertility, contraception, STI/HIV transmission, and prevention, with a greater risk perception of pregnancy and HIV compared to out-of-school girls [31]. One study [35] reported that HIV-positive individuals exhibited increased rates of risky sexual behavior [35]. Additionally, MSM and PWID were less likely than FSW to use condoms, particularly during anal sex [44].

#### Family support

Family support and socioeconomic factors significantly influence sexual behaviors, impacting the risk of STIs. Studies [38, 40] have linked risky sexual behaviors to various sociodemographic factors, including age, gender, marital status, and urban or rural residence. Those from polygamous households or with limited parental oversight showed a greater propensity for risky sexual behaviors, with approximately 71% of respondents experiencing unplanned sexual debuts [40]. The absence of parental guidance has led approximately 33% of adolescents to seek information about sex from peers or social media, highlighting a gap in parental sex education [40]. Notably, strained maternal relationships have been correlated with risky sexual behaviors [33]. Despite considerable family support, risky behaviors such as drug use, tobacco smoking, and alcohol consumption were prevalent among university students [29]. While some refrained from sexual activity, a significant portion engaged in high-risk behavior, illustrating the complexity of individual behavior and the limitations of family support alone [29]. Psychological factors such as self-efficacy, attitudes, beliefs, and mental health also intersect with family support [29].

#### Ethnic and gender influences

Cultural and gender dynamics significantly shape sexual behaviors and vulnerabilities among individuals. Females typically initiate sexual activity 16 years earlier than males [46], yet only 4.3% of females utilize female condoms, indicating low usage rates among females [45]. In contrast, while 43% of males consistently used condoms, only 16% of females did, highlighting a gender disparity in protection [46]. Similarly, approximately 41.8% of male university students used condoms consistently, compared to 34.9% of female university students [28]. This discrepancy among university students is linked to the poor sexual health knowledge and awareness observed among undergraduates [34]. The higher rate of condom usage among males stems from their greater ability to negotiate condom use [33, 46], with males often perceiving women who initiate condom discussions as promiscuous, which deters women from engaging in such negotiations [30]. Despite the higher condom usage rates among males, studies [28, 34, 37, 38, 40, 46] have reported greater risky sexual behavior, particularly multiple sex partners, among males than females. Additionally, drug use, alcohol consumption, sex enhancer drugs, and other substance abuse were more prevalent among males [29, 33, 35, 37, 44].

Significant ethnic variations in sexual behaviors were observed, underscoring the influence of cultural factors. Hausa/Fulani females aged 15–19 years and Yoruba males exhibited heightened risks of early sexual activity, reflecting cultural and gender norms [46]. Similarly, Igbo respondents were three times more likely to have multiple lifetime sexual partners than were their Yoruba and Hausa counterparts [38]. While Yoruba respondents showed a significant increase in the likelihood of having multiple partners, Igbo respondents were less likely to report early sexual debut than were Yoruba and Hausa respondents [38]. Religious beliefs also play a role in shaping sexual behaviors, as certain religious perspectives view family planning as contrary to divine will, discouraging parents from discussing sexual health with their children [27].

#### Poor sexual health intervention

Seven studies [28, 29, 33, 34, 37, 42, 44] underscored the significant role of inadequate sexual health education in fuelling the disproportionate prevalence of risky sexual behaviors. Despite the availability of sexual health interventions, promotional strategies were found to be ineffective in Ibadan, with a mere 47.9% awareness and 4.3% usage of female condoms reported [45]. Additionally, unconventional methods such as consuming stout or Andrew’s liver salt as postsex pills were observed, stemming from poor risk perception and limited awareness regarding condom benefits [25]. This pattern of inadequate risk perception contributes to the transmission of STIs [27]. Furthermore, studies [26] have highlighted a lack of understanding regarding the intricate interplay between risky behavior, HIV risk perception, and health awareness campaigns, resulting in a high prevalence of needle sharing among PWID (39.3%), thereby increasing the risk of HIV transmission [44].

### Determinants of STI risk factors

**Fig. 2:**
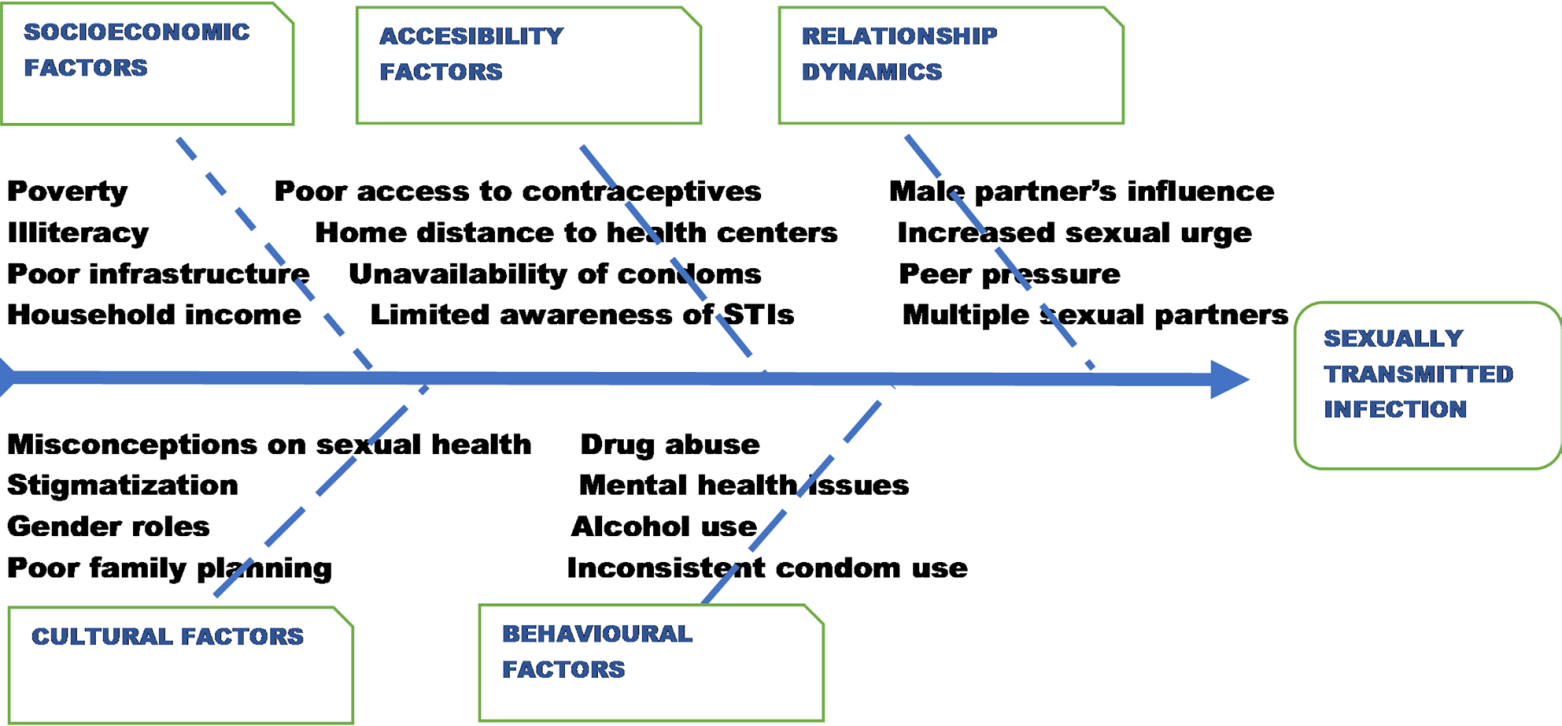
Fishbone diagram of risk factors and determinants

**Fig. 3:**
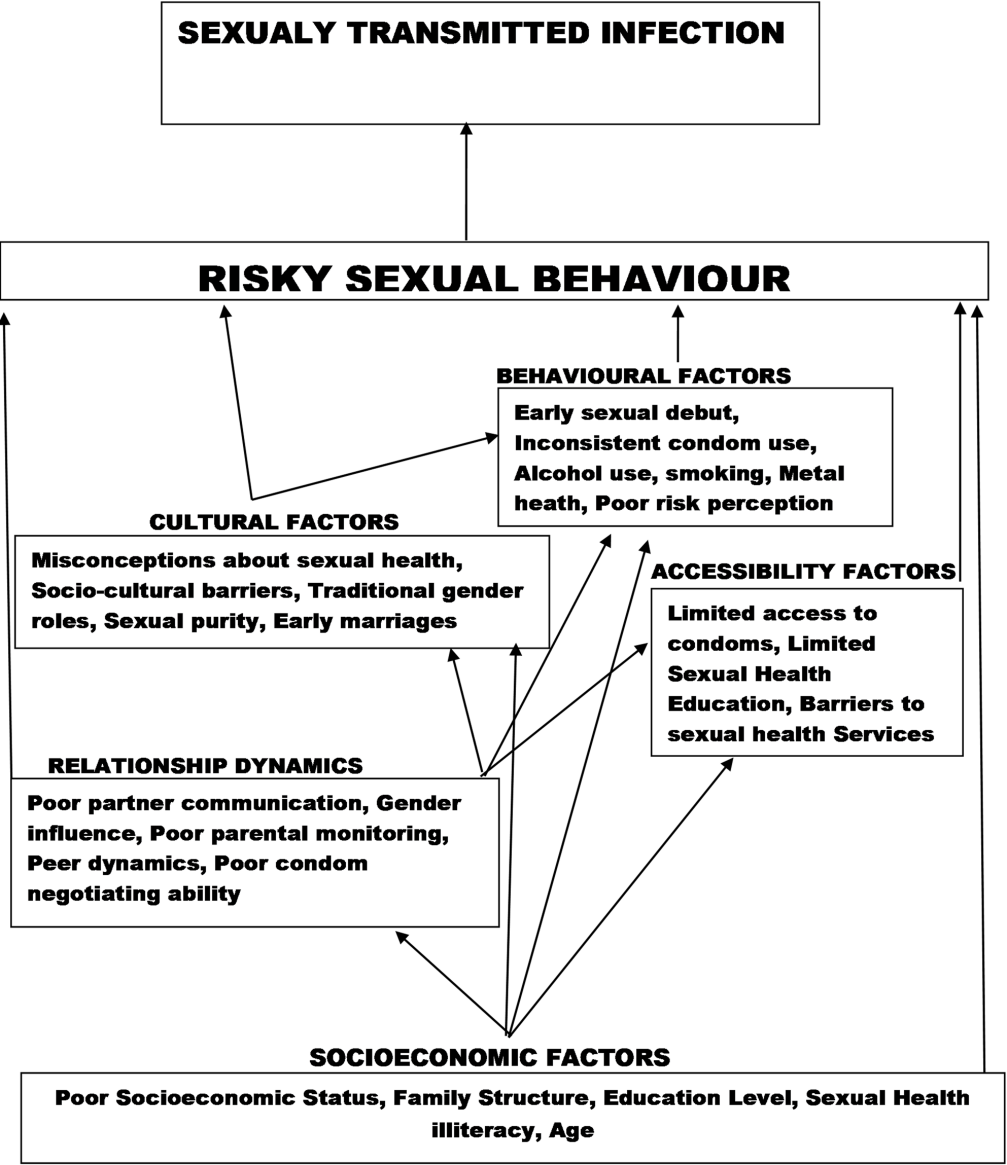
Thematic relationships associated with STIs among adolescents in Nigeria

This extensive literature review provided insights into STI risk factors in Nigeria. From 23 studies, 46 initial codes were identified, refined into 16 subthemes, and synthesized into five major themes: socioeconomic, cultural, accessibility, relationship dynamics, and behavioral factors (Table 2). Socioeconomic elements such as education and employment impact contraceptive use and sexual health outcomes. Cultural norms and gender roles influence behaviors, while limited access to sexual health interventions and relationship dynamics pose challenges (Fig. 2). Relationships among themes revealed that limited access to sexual health interventions and contraceptives, driven by socioeconomic disparities, educational gaps, and cultural norms, are the main barriers to healthy sexual behaviors in Nigeria (Fig. 3). Despite awareness of STIs and condoms, actual use remained low due to economic constraints, misconceptions, gender disparities in contraceptive decision-making, health-seeking behaviors, risk perception, and substance abuse. These findings highlight the complexity of sexual health determinants and the need for comprehensive interventions.

**Table 2:** Determinants of risk factors associated with STIs in Nigeria

Themes	Sub themes	Codes	Reference
1. Socioeconomic Factor	Education Level	Secondary Education	[25, 32, 36, 38, 43]
		Tertiary Education	[28, 37]
	Employment and Socioeconomic status	Employment Status	[38]
		Socioeconomic Status	[38, 42]
	Sexual Health Education	Awareness of STI and Healthy Sexual Behavior	[25, 37, 43]
	Socio-Demographic Factors	Family Structure	[27, 36, 40, 43]
		Gender	[28, 46]
		Age	[32, 38]
2. Cultural Factor	Beliefs and Misconceptions	Misconceptions driving risky sexual behaviors	[25, 37]
	Sociocultural Barriers	Sociocultural perceptions on family planning practices.	[25, 27, 28, 30, 44, 46]
		Traditional gender roles	[38, 46]
	Cultural Influences on Sexual Behavior	Ethnicity	[27, 38, 46]
		Early Marriages	[31, 43, 46]
		Polygamy	[36, 46]
		Sexual Purity	[46]
3. Accessibility Factor	Access to Contraceptives	Poor access to condoms	[25, 27]
		Unavailability of condoms	[28, 43]
	Sexual Health Education	Limited Knowledge and awareness of STIs	[37, 39, 41]
		Limited sexual health education	[37, 39]
		Limited Knowledge and awareness of condom	[37, 39]
		Lack of sexual health education in schools	[32, 37]
		Inadequate sexual health education in families	[25, 39]
		Limited sexual health education in communities	[25, 27]
	Access to Sexual Health Interventions	Inadequate reproductive health services	[27]
		Limited sexual health infrastructure	[26, 44]
		Disparities in sexual health interventions	[27, 37]
4. Relationship Dynamics	Gender influence in Partner Communication	Influence of male partners	[29, 30]
		Perceptions on condom use	[28, 46])
		Poor Contraceptive/condom negotiating ability	[29, 44, 46]
	Supportive Relationships	Family support in protective sexual behaviors	[29, 40]
		Parental monitoring	[36]
		Household structure influence on risky behaviors	[36]
		Marital status	[42]
		Peer pressure	[25, 26]
5. Behavioral Factor	Poor sexual health decision	Early sexual debut	[32]
		Increased sexual urges for adolescents	[31]
	Condom Use	Inconsistent condom use	[25, 29]
	Substance Use	Substance use	[33, 44]
		Smoking	[33]
		Alcohol use	[33]
		Sex enhancer drug use	[37]
	Health Seeking behavior	Poor self-efficacy	[28]
		Poor STI knowledge	[31, 34, 42, 43]
		Low condom use	[28, 42, 45, 46]
		Mental health issues	[39]
		Poor risk perception	[27]

### Socioeconomic factors

#### The role of education

We observed that education significantly shaped sexual behaviors and contraceptive knowledge, with secondary education being particularly crucial. Primary or secondary education reduces unprotected sex [38, 43]. However, secondary school adolescents in southwestern Nigeria still engage in risky behaviors such as early sexual debut and inconsistent condom use [32]. While secondary school students knew about condom use for STI prevention, their actual usage was low [25]. High-risk behaviors among Hausa/Fulani girls in northern Nigeria were linked to educational discrimination [46]. Maternal education predicted the sexual behavior of individuals, with more educated mothers providing better sexual health support [36]. Husbands’ education is positively correlated with family planning use [46]. Few university students consistently use condoms, with self-efficacy and HIV discussions improving use [28]. High-risk behaviors among undergraduates were blamable to poor sexual health education at the tertiary level [37].

#### Employment and socioeconomic status

Employment and socioeconomic status significantly impact contraceptive use and sexual health outcomes. Employment status predicts risky sexual behaviors, with those employed being more willing to purchase condoms [38, 43]. Higher socioeconomic status is correlated with better sexual health outcomes and promotes healthy sexual behavior [39]. Modern contraceptive use is influenced by the socioeconomic status of households [18]. Individuals from families without a lively hood or with a lower socioeconomic status have less awareness of sexual health and less access to modern contraceptives.

#### Sexual health education

The level of sexual health education and awareness significantly affects sexual behavior. Risky sexual behaviors, particularly inconsistent condom use, are linked to significant knowledge gaps [25, 43]. Additionally, disparities in sexual health knowledge between in-school and out-of-school adolescents are also notable. Compared with their out-of-school peers, in-school girls have better knowledge of sexual and reproductive health [37].

#### Sociodemographic factors

Young individuals from polygamous homes and those with unmarried parents were more likely to experience a lack of parental monitoring, leading to increased engagement in risky behaviors [40]. Age significantly influences behavior, with younger individuals being more prone to early sexual debut and inconsistent condom use [32, 43]. Adolescents aged 14–16 years exhibited lower exposure to risk factors than did those aged 17–19 years [38]. Additionally, two studies [28, 46] reported gender differences in condom usage, with males demonstrating higher usage rates than females.

### Cultural factors

#### Beliefs and misconceptions

Some individuals held significant misconceptions about condoms, including beliefs that improper use could cause death and that condoms could be washed and reused [25]. Additionally, some believe that condoms have no benefits and can actually increase the risk of STIs and unintended pregnancies [26]. There are also misconceptions about condoms permanently affecting male fertility [25]. These erroneous beliefs led individuals to use incorrect alternatives, such as hard drugs and laxatives, to prevent pregnancy [25, 26]. Such misconceptions contribute to risky sexual behaviors such as early sexual debut and inconsistent condom use among adolescents [26, 37].

#### Gender barriers

Gender barriers significantly influenced access to sexual health resources and behaviors. Studies [27, 30] have indicated that gender affects contraceptive use among rural Southeast Nigerian women and couples, with male partners often viewing condom use as promoting promiscuity, leading to a preference for natural methods over modern contraceptives. Conversely, males exhibited greater condom use, reflecting gender norms that prioritize male sexual health [28, 46]. Compared with FSW, lower condom use rates were found among MSM and PWID [44]. One study [47] noted an increase in condom use among parous women. Despite progress, traditional beliefs continued to hinder consistent condom use, with taboo perceptions and limited open communication perpetuating misconceptions and risky behaviors [25].

#### Ethnic influences on sexual behavior

Ethnicity significantly influenced sexual behavior among young Nigerians, reflecting diverse cultural norms. Risky sexual behavior was more prevalent in polygamous communities, where male superiority fostered entitlement to multiple partners [46]. Ethnic differences also affected the reporting of sexual behaviors; Individuals from the Igbo ethnicity were less likely to report early sexual debut than were their Yoruba and Hausa peers. Conversely, Hausa/Fulani females face greater risks of early sexual debut due to educational discrimination and early marriages [46]. Yoruba and Igbo males exhibited greater condom use and multiple sexual partnerships than did their Hausa counterparts [46].

### Accessibility factors

#### Unavailability of condoms

Access to condoms remained a significant challenge, as highlighted by the reviewed studies. Poor access to sexual health interventions and contraceptives is prevalent in many regions. For instance, married women in rural Southeast Nigeria had low condom use due to the unavailability of modern options such as condoms [27], indicating a critical gap in condom distribution and accessibility, especially in rural areas. Additionally, the shortage of condoms was a pressing issue. Fewer than half of the university students consistently used condoms, partly due to availability problems [28]. This shortage could lead to condom reuse [25], increasing the risks of STI transmission and unintended pregnancies, particularly among sexually active individuals.

#### Poor awareness

While knowledge and awareness of STI promoted healthy sexual behaviors [39], limited sexual education from diverse sources such as schools, families, and media was accessible [41], and depending only on schools might have overlooked certain groups. The deficiency in comprehensive sexual education within schools, despite their potential for broader awareness [32, 37], is concerning and potentially contributes to risky behaviors among secondary school students [32] and undergraduates [37]. Additionally, family-based education perpetuated misinformation due to cultural taboos [40]. Exposure to harmful content on social media [25] also risked reinforcing misconceptions about condom use.

#### Access to sexual health interventions

Insufficient access to reproductive health services poses significant barriers. Women in Rural Southeast Nigeria, for instance, face challenges accessing condoms due to socioeconomic barriers such as the distance to health facilities [27]. Moreover, socioeconomic disparities exacerbate the situation, with the absence of sexual health clinics [26, 44] and, where present, inadequate conditions [42], contributing to a lack of knowledge about STI transmission and limited access to sexual health interventions. This issue is further compounded by geographic and economic factors, particularly impacting rural areas [26, 46].

### Relationship dynamics

#### Condom negotiating ability and partner communication

The influence of having male partners on sexual health decisions was significant. The perceptions of male partners impacted contraceptive use among Nigerian couples, leading women to adhere to their partners’ preferences, sometimes at their own expense [30]. Male involvement in sexual health, particularly regarding perceptions of contraceptive use, was crucial. Men often control condom use decisions, making them more inclined to report usage compared to women [46]. Power dynamics within relationships affect contraceptive and condom negotiation abilities. Less than half of university students consistently use condoms due to poor negotiation skills influenced by power imbalances [28]. Similarly, inconsistent condom use among MSM, FSW, and PWID stems from poor negotiation abilities. Additionally, challenges were observed for young females negotiating condom use with older partners [46].

#### Supportive relationships

Supportive family and partner relationships play a pivotal role in promoting healthy sexual behaviors [29]. Strong family support and parental monitoring are associated with safer sexual practices and reduced engagement in risky behaviors [26]. Parental discussions about sexual health topics equip young individuals with essential knowledge [40] and foster responsible behavior [36]. This parental involvement was significantly influenced by household structure, with individuals from polygamous homes or those lacking parental figures being more prone to risky sexual activities [36, 40]. Compared with their married counterparts, unmarried women face distinct challenges in supporting their children with sexual health interventions [42]. However, peer pressure significantly influences eccentric and dangerous pregnancy prevention methods [26], encouraging risky sexual behaviors among [25]. Certain individuals particularly the young adults often succumb to peer norms, leading to the adoption of risky behaviors such as drug abuse [29, 33] and early sexual debut [32, 37, 38]. Peer-influenced misconceptions further exacerbate misunderstandings, contributing to a culture of misinformation surrounding condom use [26, 33].

### Behavioral factors in sexual health

#### Sexual health decisions

The risk factors for STIs, particularly among adolescents in southwestern Nigeria, were influenced by poor sexual health decisions. Early sexual debut and drug abuse are prevalent among secondary school adolescents, increasing their risk of contracting STIs [32]. Furthermore, increased sexual urges among individuals led them to seek alternatives to condoms due to peer pressure and financial constraints [31]. Peers often promote unconventional methods such as consuming alcoholic drinks and Andrew’s liver salt to flush out pregnancy, thereby exacerbating risky behaviors and contributing to the prevalence of STIs [25].

#### Inconsistent condom use

Inconsistent condom use has remained a significant challenge [28]. This inconsistency is often attributed to a lack of negotiation skills [28, 44] and is perpetuated by misconceptions surrounding condom use [25, 28, 30, 44, 46]. Moreover, availability barriers hinder willingness to use condoms regularly [28, 43]. Furthermore, disparities in condom use exist among different demographic groups, including FSW, MSM, and PWID [44]. Addressing behavioral barriers, such as improving negotiation skills and dispelling misconceptions, was essential for promoting consistent condom use and preventing STI transmission.

#### Substance abuse

Substance abuse, including smoking, alcohol consumption, and the use of sex-enhancing drugs, significantly influenced engagement in risky sexual behaviors. The consumption of psychoactive substances impaired the ability to negotiate condom use, leading to a greater frequency of unprotected sexual encounters due to diminished inhibition. This increased the likelihood of contracting STIs and unintended pregnancies [44]. In a study conducted in Osun State, alcohol and drug use emerged as significant contributors to risky sexual behaviors among university undergraduates, with the university environment exacerbating these issues and normalizing such behaviors [33]. Additionally, the widespread use of sex-enhancing drugs among was associated with a decrease in condom usage, contributing to long-term health and socioeconomic consequences, including incurable complications from STIs, early pregnancy, and school dropout [37].

#### Health seeking behavior

Poor self-efficacy and limited discussions about STIs often result in inadequate health-seeking behaviors. One study [28] showed that higher self-efficacy levels and consistent discussions on STIs positively influence condom use consistency. Despite being aware of STIs, many individuals still exhibit poor self-efficacy and avoid discussions about STIs, leading to low rates of STI testing [31, 42, 43]. Nonetheless, limited knowledge about STIs also influences health-seeking decisions, particularly among polytechnic students, contributing to low condom use rates [34]. Interestingly, while females generally demonstrated greater sexual health awareness than males did, both genders still exhibited low rates of condom use [31, 42, 43]. However, there was a positive correlation between female condom awareness and consistent condom usage [45].

#### Sexual risk perception and protection

Mental health issues frequently intersect with risky sexual behaviors, as individuals experiencing poor mental health often underestimate the consequences of their actions [39]. Exacerbation of poor mental health heightened vulnerabilities such as low self-esteem or depression, impeding informed decision-making and increasing susceptibility to adverse sexual health outcomes [39]. In rural communities lacking sexual health infrastructure, residents often exhibit poor risk perception, leading to increased transmission rates of STIs due to limited awareness and inconsistent condom use [27]. Additionally, individuals often resort to unconventional methods such as consuming stout or Andrew’s liver salt postsex to attempt to prevent pregnancy, indicating a form of poor risk perception [25]. Excessive alcohol and drug abuse further compound poor mental health, contributing to risky sexual behaviors among undergraduates [33]. This impaired judgment frequently fueled the use of sex enhancer drugs, engagement in pornography, and other substance abuse, thereby heightening vulnerability to STIs and unintended pregnancies. Consequently, addressing mental health alongside sexual health interventions is imperative for comprehensive outcomes.

## Discussion

Our systematic review shifted from the simplistic view of risk factors associated with STIs among Nigerians to explore the multifaceted factors influencing sexual health in Nigeria. Drawing on public health theories [48], our study identified socioeconomic status, cultural norms, accessibility, relationship dynamics, and behaviors as critical in shaping STI risk factors. Socioeconomic factors, influenced by SDHs, educational gaps, and cultural norms, significantly impede access to sexual health interventions and contraceptives among Nigerians particularly adolescents who are considered deprived. Our review underscores the critical barrier of limited access to these services, rooted in socioeconomic inequalities and exacerbated by the EMHP. Economic constraints often prevent individuals from receiving contraceptives, perpetuating risky sexual behaviors.

Despite widespread awareness about STIs and condoms, actual condom use among remains low compared to other populations studied, revealing a stark gap between knowledge and practice highlighted by the HBM. Misconceptions about condom use, particularly among culturally influenced populations, further hinder uptake. Gender disparities, viewed through the lens of SDH, also play a significant role, with male partners often dominating contraceptive decisions, thereby restricting women’s autonomy. Power imbalances within relationships, influenced by PIT, compromise the ability to negotiate condom use effectively, contributing to unsafe sexual practices. Conversely, supportive family and healthy partner relationships emphasized by the PIT and the HPM foster healthier sexual behaviours across age groups. Inadequate self-efficacy, as examined through the HBM and limited discussions about STIs contribute to insufficient health-seeking behaviours. Furthermore, mental health issues, considered within EMHP, further complicate vulnerabilities, impairing decision-making related to sexual health. The ineffective implementation of sexual health policies crucially hampers condom distribution and accessibility, particularly in rural and underserved areas, aligning with recommendations from the HPM.

We noted that limited access to targeted sexual health interventions and condoms across diverse age group in Nigeria is primarily influenced by economic constraints, posing significant challenges for individuals seeking reproductive healthcare. Our finding is consistent with surveys conducted by [49] among marginalized populations, such as adolescent girls or low-income families, which revealed that financial barriers prevented individuals from purchasing contraceptives or seeking reproductive healthcare. Economic disparities result in unequal access to essential services, with marginalized populations often bearing the brunt of these limitations. For instance, in rural areas where poverty rates are higher, individuals may struggle to afford contraceptives or access healthcare facilities that provide them. This economic barrier not only restricts individuals’ ability to obtain condoms but also perpetuates a cycle of limited reproductive autonomy and heightened vulnerability to unintended pregnancies and STIs. In contrast, where there is willingness and purchasing power, the unavailability of condoms worsens the situation, particularly in remote regions where healthcare infrastructure is underdeveloped. This finding is consistent with the findings of another systematic review in rural areas in sub-Saharan Africa [18], which highlighted the limited availability of healthcare facilities and trained personnel, resulting in inadequate access to contraceptive services. In such areas, individuals may lack access to basic reproductive health services, including contraception counseling and distribution. This absence of essential resources not only hampers individuals’ ability to make informed decisions about their sexual health but also forces them to resort to risky alternatives or forego contraception altogether. Consequently, this contributes to a higher prevalence of unplanned pregnancies and STIs among populations with limited access to sexual health interventions. The gap between condom knowledge and usage highlights the intricate link between awareness and accessibility. Despite education and awareness on contraceptive, its practical utilization remains minimal and is hindered by barriers such as stigma. Cultural norms and religious beliefs perpetuate myths about safety and effectiveness, discouraging use. Misconceptions about side effects and interference with fertility deter adoption. Traditional practices favoring fertility over contraception, particularly for women, impede access. However, it is crucial to acknowledge the strides made in addressing these challenges and enhancing access to contraceptives particularly among marginalized young populations. Initiatives such as Youth-Friendly Health Services prioritize confidentiality and tailor services to young people’s needs [50].

In addition, sexual health education programs empower peers to conduct workshops and outreach, fostering knowledge sharing among sexually active individuals [51]. Mobile health initiatives, including SMS hotlines and apps, offer discreet advice on contraceptive use, complementing school-based health education, which equips individuals with essential reproductive health knowledge [52]. Another systematic review [53] highlighted that, concerted efforts, coupled with mass media campaigns via radio dramas, TV ads, and social media, help in combating stigma, enhancing awareness, and breaking down economic and cultural obstacles that impede access to contraceptives, even in rural and underserved regions.

We highlighted that gender disparities in condom decision-making perpetuate unequal power dynamics within relationships in Nigeria. According to the findings of [54], traditional gender norms often dictate men as primary decision-makers in sexual activities with male partners commonly exert control over condom use, leaving women with limited autonomy in reproductive health decisions. Women feel pressured to adhere to their partner’s preferences, compromising their own reproductive goals and health needs. Previous qualitative studies [55] and reviews [56, 57] have shed light on the prevalence of condom coercion and male-dominated decision-making within relationships. The power dynamics in relationships often compel women to defer to their partners’ choices due to fear of reprisal. This situation hinders their ability to negotiate condom use and undermines their rights and ability to make informed choices, exacerbating gender disparities in sexual health decision-making. This leaves women vulnerable to unprotected sex, STIs, and unintended pregnancies. Contrary to our findings, a systematic review by [58] contends that initiatives promoting gender equality empower women to assert their reproductive rights and negotiate contraceptive use within their relationships.

Supportive family and partner relationships are pivotal in shaping engagement in healthy sexual behaviors. These relationships foster open communication, enabling young adults to discuss sexual health topics comfortably. Familial support serves as a protective factor against risky sexual activities by providing comprehensive sexual education and guidance. Similarly, supportive partner relationships encourage responsible sexual behavior through mutual respect, open communication, and shared decision-making. Consistent with our findings, previous studies have shown that young individuals with supportive and communicative relationships with their parents or guardians are more likely to delay sexual debut, use contraception consistently, and engage in fewer risky sexual behaviors [53, 59–61]. Conversely, individuals who lack supportive family or partner relationships due to factors such as family dysfunction, parental neglect, or unhealthy romantic relationships may experience barriers to accessing sexual health information and resources, increasing their vulnerability to engaging in risky sexual behaviors [62, 63]. Overall, supportive family and partner relationships are crucial for the sexual health of individuals, emphasizing the need for positive communication within families and among romantic partners to empower informed decision-making and promote safe sexual practices [64].

We highlighted poor self-efficacy and limited discussions about STIs as significant implications for health-seeking behaviors and sexual health outcomes. Studies have shown that when individuals lack confidence in their ability to seek and access sexual health services, they are less likely to take proactive measures, such as consistent condom use, regular STI testing, and seeking medical advice for sexual health concerns, to protect themselves from STIs and other sexual health risks [65, 66]. Additionally, limited discussions about STIs can lead to a lack of awareness and understanding of the importance of STI testing and condom use in preventing transmission [67]. A systematic review by [68] noted that low rates of STI testing and condom use due to inadequate health-seeking behaviors lead to delayed diagnosis and treatment of STIs, potentially resulting in complications. Without regular testing, individuals may unknowingly transmit infections to their partners [69]. Additionally, our findings, which underscore that a lack of confidence in negotiating condom use led to more frequent unprotected sex, align with the findings of a qualitative study on condom use [70]. The qualitative study noted that a lack of awareness of the importance of condoms in preventing STIs predicts negotiating abilities, thereby endangering both individuals and their partners. Scaling up testing and condom use, considering poor self-efficacy and health-seeking behavior, requires implementing ethical incentives and behavioral change approaches similar to those used in genitourinary clinics in England [71]. These initiatives can empower individuals by incentivizing workshop participation, offering confidential counseling, and fostering a supportive environment for behavioral change. Tackling poor self-efficacy and limited STI discussions is vital for promoting proactive health-seeking behaviors and reducing STI transmission.

Our review revealed that mental health issues, including those related to sexual health, profoundly influence individuals’ decision-making processes. When individuals experience mental health challenges such as depression, anxiety, or low self-esteem, their cognitive functioning and judgment may be impaired, leading to difficulties in making informed decisions about sexual health. Similarly, previous research [72] indicated that mental health issues are correlated with a greater likelihood of engaging in risky sexual behaviors. Individuals with depression or anxiety are more prone to report inconsistent condom use, having multiple sexual partners and substance abuse during sexual encounters than are those without mental health issues [39]. This is because individuals with mental health issues may struggle with reduced risk perception and evaluating consequences [27]. Furthermore, mental health issues can exacerbate vulnerabilities, increasing the likelihood of engaging in risky sexual behaviors. This is particularly relevant among key populations such as MSM, FSW, and PWID. Individuals in these groups dealing with depression or low self-esteem may turn to sexual activity for validation or temporary relief from emotional distress, even when it involves risks such as unprotected sex exacerbated by substance abuse [73–75]. Addressing the impact of mental health on sexual health decision-making is crucial for positive outcomes. Integrated interventions can enhance coping strategies and self-efficacy, enabling informed decisions about sexual activity [76]. Integrating mental health services into sexual health programs and offering comprehensive support for individuals with mental health issues can mitigate negative effects on sexual health decision-making [77, 78].

### Implication for public health

Our findings have several key public health implications for Nigeria. Comprehensive sexual education programs are urgently needed to address knowledge gaps and misconceptions about contraceptives, STI prevention, and healthy relationships. Tailored interventions involving schools, families, and media, including the use of social media, can effectively disseminate information. Public health campaigns should debunk myths about condoms and contraceptives, provide evidence-based information, and address gender barriers. Ensuring the availability of condoms, particularly in rural areas, and offering free or subsidized options can improve accessibility. Comprehensive sexual health services that prioritize confidentiality, counseling, and support are essential for improving safe sexual practices. Integrating comprehensive sexual health education with practical access to contraceptives, as advocated by the Ecological Model of Health Promotion, was recommended to bridge the gap between knowledge and behavior. Additionally, parental involvement in sexual health education can positively influence sexual health behaviors of young adults. Special programs should educate and encourage parents on discussing sexual health topics and address the needs of individuals from polygamous homes or those with inadequate parental monitoring. Community leaders and peer educators can help overcome cultural barriers and promote positive behaviors. Targeted interventions are needed to address the high prevalence of substance abuse and its impact on sexual health. Schools and higher education institutions should educate students on the risks of substance abuse and promote healthy lifestyle choices. Enhancing self-efficacy and communication skills related to condom negotiation can empower individuals to make safer sexual decisions. Ultimately, policymakers should implement evidence-based policies addressing risky sexual behaviors and regularly monitor and evaluate sexual health programs. An integrated approach combining education, accessibility, family support, and behavioral interventions is crucial. Unless there is collaboration among government agencies, NGOs, and international partners, the effectiveness and sustainability of sexual health interventions may be compromised, hindering efforts to reduce STIs and unintended pregnancies particularly among young individuals.

### Limitations of the study

Our review has several strengths. It is nationally representative, including studies from both rural and urban areas across Nigeria’s six geopolitical zones. The inclusion of both qualitative and quantitative data enriched our analysis, providing a well-rounded understanding of the issues. Importantly, the review captured multiple age groups reflecting adolescents, young adults, and broader adult populations, which enhances the applicability of the findings across diverse demographic segments. The interdisciplinary approach, incorporating history, sociology, and public health, enhances the depth and relevance of our findings, strengthening the validity of our conclusions and broadening their impact. However, we identified several limitations. Five articles [30, 38, 39, 42, 46] relied on survey data, limiting the depth and context of the findings. Self-reported data introduce potential bias, such as underreporting or over reporting of sexual behaviors and contraceptive use. Variations in sample size and diversity across studies could affect the robustness of our conclusions, with smaller samples providing weaker evidence. All included studies except study [26] employed a cross-sectional design, limiting our ability to establish causality and only identify associations. The heterogeneity of the studies prevented a quantitative meta-analysis. Additionally, our review disproportionately focused on the southern region of Nigeria [25–27, 32–34, 36, 37, 40, 41, 44, 45, 47]. This geographical imbalance constrains the broader applicability and generalizability of the findings, leaving the northern region underrepresented. Nonetheless, more than 26% [26, 30, 38, 39, 42, 46] of the articles reviewed were national representatives.

## Conclusion

This review revealed a complex web of determinants influencing sexual health risk factors in Nigeria. Economic constraints significantly limit access to contraceptives, amplifying risky sexual behaviors and adverse outcomes. Gender disparities in contraceptive decision-making highlight the need for interventions promoting women’s autonomy and equitable partnerships. Supportive family relationships are crucial in shielding against risky behaviors and fostering responsible sexual health practices. Low self-efficacy and inadequate discussions about STIs result in low rates of STI testing and condom use, posing public health challenges. Mental health issues further complicate decision-making, emphasizing the need to integrate mental health support into sexual health interventions. Addressing these challenges requires strategic policy interventions. Prioritizing contraceptive distribution, integrating sexual health education with accessible services, and closing the knowledge-practice gap through collaboration are essential steps to improve sexual health outcomes in Nigeria.

## Supplementary Information

Supplementary Material 1.

## Data Availability

Data and materials used in this review are available from the corresponding author on reasonable request.

## Abbreviations

AJOL: African Journals Online
AIDS: Acquired Immune Deficiency Syndrome
CDC: Centers for Disease Control and Prevention
DHS: Demographic and Health Survey
EMHP: Ecological Model of Health Promotion
EPKI: Edobor Peter Kenneth Imarenezor
FSW: Female Sex Workers
HBM: Health Belief Model
HIV: Human Immunodeficiency Virus
HPM: Health Promotion Model
HW: Health Worker
JBI: Joanna Briggs Institute
LMICs: Low–and Middle–Income Countries
MICS: Multiple Indicator Cluster Survey
ML: Mixed–Methods (if you used this shorthand)
MSM: Men Who Have Sex with Men
NDHS: Nigeria Demographic and Health Survey
NACA: National Agency for the Control of AIDS
PIT: Parental Involvement Theory
PLHIV: People Living with HIV (if used–appears indirectly)
PRISMA: Preferred Reporting Items for Systematic Reviews and Meta–Analyses
PROSPERO: International Prospective Register of Systematic Reviews
PWID: People Who Inject Drugs
SDH: Social Determinants of Health
SDP: Service Delivery Point
SRH: Sexual and Reproductive Health
STI: Sexually Transmitted Infection
UN: United Nations (if referenced)
WHO: World Health Organization

## References

[1] Fasciana T, Capra G, Lipari D, Firenze A, Giammanco A. Sexually transmitted diseases: Diagnosis and control. Int J Environ Res Public Health. 2022;19(9):5293. 10.3390/ijerph19095293. 35564688 10.3390/ijerph19095293 PMC9105465

[2] WorldHealth Organization (WHO). Sexually transmitted infections (STIs). 2025. https://www.who.int/news-room/fact-sheets/detail/sexually-transmitted-infections-%28stis%29.

[3] James C, Harfouche M, Welton NJ, et al. Herpes simplex virus: global infection prevalence and incidence estimates, 2016. Bull World Health Organ. 2020;98(5):315–29. 10.2471/BLT.19.237149. 32514197 10.2471/BLT.19.237149 PMC7265941

[4] Unemo M, Lahra MM, Escher M, Eremin S, Cole MJ, Galarza P, Ndowa F, Martin I, Dillon JR, Galas M, Ramon-Pardo P, Weinstock H, Wi T. WHO global antimicrobial resistance surveillance (GASP/GLASS) for Neisseria gonorrheae 2017–2018: a retrospective observational study. Lancet Microbe. 2021;2. 10.1016/S2666-5247(21)00171-3. 10.1016/S2666-5247(21)00171-3 35544082

[5] Dias SP, Brouwer MC, van de Beek D. Sex and gender differences in bacterial infections. Infect Immun. 2022;90. 10.1128/iai.00283-22. 10.1128/iai.00283-22 PMC9584217 36121220

[6] Mofolorunsho KC, Dorsamy V, Bagwandeen C, et al. Prevalence of gonococcal and chlamydial infections among men who have sex with men in sub-Saharan Africa: protocol for a systematic review and meta-analysis. Syst Rev. 2023;12:141. 10.1186/s13643-023-02305-2. 37580787 10.1186/s13643-023-02305-2 PMC10424383

[7] Keller L. Reducing STI cases: Young people deserve better sexual health information and services. Age. 2020;79(65):65. https://www.guttmacher.org/sites/default/files/article_files/gpr2300620.pdf.

[8] NACA. (2016). National HIV Strategy For Adolescents and Young People. – NACA Nigeria https://naca.gov.ng/national-hiv-strategy-adolescents-young-people.

[9] Owowo EE, Udofia LE, Wisdom S, Okon IE. Incidence of Trichomonas vaginalis among internally displaced women in Ibaka, Akwa Ibom State, Nigeria. J Biosci Med. 2022;10(3):82–9. 10.4236/jbm.2022.103009.

[10] Asemota OO. Trichomoniasis in Nigeria: A review. Biomed Res. 2018;29(12):2532–9. 10.4066/biomedicalresearch.29-18-493.

[11] Eguvbe AO, Alex-Wele MA, Abdu AB, Egbagba JE. Genital Herpes: Synergy between Serology and Polymerase Chain Reaction in Laboratory Diagnosis among Pregnant Women Attending the Antenatal Clinic of Federal Medical Centre, Yenagoa Nigeria. Int STD Res Reviews. 2021;10(1):31–8. 10.9734/ISRR/2021/v10i130121.

[12] Ferenc MV, Šerman A, Bekavac I, Vasilj O. Characteristics of Acute Pelvic Inflammatory Disease in Surgically Treated Females Over Ten Years-A Single-Center Study. Curr Women’s Health Reviews. 2024;20(3):65–73. 10.2174/1573404820666230518103039.

[13] Saha U. Neonatal Mortality and Morbidity: The Burden. In Clinical Anesthesia for the Newborn and the Neonate (pp. 3–10). Singapore: Springer Nature Singapore. 10.1007/978-981-19-5458-0_1.

[14] Mabuza MP, Mabuza MP, Disease Control and the Promotion of Public Health Equity. Evaluating International Public Health Issues. : Critical Reflections on Diseases and Disasters, Policies and Practices. 2020:105–254. 10.1007/978-981-13-9787-5_5.

[15] Al-Worafi YM. Epidemiology and Burden of Infectious Diseases in Developing Countries: HIV and STIs. InHandbook of Medical and Health Sciences in Developing Countries: Education, Practice, and Research 2023 Dec 15 (pp. 1–22). Cham: Springer International Publishing. 10.1007/978-3-030-74786-2_340-1.

[16] Alawode OA, Ogunwemimo H, Bolorunduro ME, Awoleye AF. Age at sexual debut and multiple sexual partnerships among adolescents in Nigeria: an assessment of the mediating role of the knowledge of sexually transmitted infections. Adolescents. 2021;1(4):421–32. 10.3390/adolescents1040032.

[17] Amare T, Yeneabat T, Amare Y. A Systematic Review and Meta-Analysis of Epidemiology of Risky Sexual Behaviors in College and University Students in Ethiopia, 2018. J Environ public health. 2019;2019(1):4852130. 10.1155/2018/7375831. 31015844 10.1155/2019/4852130 PMC6446110

[18] Ninsiima LR, Chiumia IK, Ndejjo R. Factors influencing access to and utilization of youth-friendly sexual and reproductive health services in sub-Saharan Africa: a systematic review. Reproductive health. 2021;18:1–7. 10.1186/s12978-021-01183-y. 34176511 10.1186/s12978-021-01183-y PMC8237506

[19] Arije O, Hlungwani T, Madan J. Key informants’ perspectives on policy- and service-level challenges and opportunities for delivering adolescent and youth-friendly health services in public health facilities in a Nigerian setting. BMC Health Serv Res. 2022;22:1493. 10.1186/s12913-022-08860-z. 36476291 10.1186/s12913-022-08860-z PMC9727905

[20] Akinyetun T. Youth Political Participation, Good Governance and Social Inclusion in Nigeria. Commonw Youth Dev. 2020;18(2). https://hdl.handle.net/10520/ejc-cydev-v18-n2-a8

[21] Ruggeri KA, Benzerga A, Verra S, Folke T. A behavioral approach to personalizing public health. Behav Public Policy. 2023;7(2):457–69. 10.1017/bpp.2020.31.

[22] Moher D, Liberati A, Tetzlaff J, Altman DG, PRISMA Group. Preferred reporting items for systematic reviews and meta-analyses: the PRISMA statement. PLoS Med 6(7):e1000097. 10.1371/journal.pmed.1000097. 10.1371/journal.pmed.1000097 PMC2707599 19621072

[23] Munn Z, Barker TH, Moola S, Tufanaru C, Stern C, McArthur A, et al. Meth¬odological quality of case series studies: an introduction to the JBI critical appraisal tool. JBI Database Syst Rev Implement Rep. 2019;2127–33. 10.11124/JBISRIR-D-19-00099. 10.11124/JBISRIR-D-19-00099 33038125

[24] Corbin JM, Strauss A. Grounded theory research: Procedures, canons, and evaluative criteria. Qual Sociol. 1990;13:3–21. 10.1007/BF00988593.

[25] Mbachu CO, Agu IC, Obayi C, Eze I, Ezumah N, Onwujekwe O. Beliefs and misconceptions about contraception and condom use among adolescents in south–east Nigeria. Reproductive Health. 2021;18:1–8. 10.1186/s12978-020-01062-y. 33407642 10.1186/s12978-020-01062-y PMC7789795

[26] Folayan MO, Sam-Agudu NA, Harrison A. Exploring the why: risk factors for HIV and barriers to sexual and reproductive health service access among adolescents in Nigeria. BMC Health Serv Res. 2022;22:1198. 10.1186/s12913-022-08551-9. 36151543 10.1186/s12913-022-08551-9 PMC9508705

[27] Akamike IC, Madubueze UC, Okedo-Alex IN, et al. Perception, pattern of use, partner support and determinants of uptake of family planning methods among women in rural communities in Southeast Nigeria. Contracept Reprod Med. 2020;5:14. 10.1186/s40834-020-00120-x. 32884833 10.1186/s40834-020-00120-x PMC7461266

[28] Ajayi AI, Ismail KO, Akpan W. Factors associated with consistent condom use: a cross-sectional survey of two Nigerian universities. BMC Public Health. 2019;19:1207. 10.1186/s12889-019-7543-1. 31477068 10.1186/s12889-019-7543-1 PMC6719351

[29] Ajayi AI, Okeke SR. Protective sexual behaviors among young adults in Nigeria: influence of family support and living with both parents. BMC Public Health. 2019;19:983. 10.1186/s12889-019-7310-3. 31337383 10.1186/s12889-019-7310-3 PMC6651974

[30] Blackstone SR, Iwelunmor J. Determinants of contraceptive use among Nigerian couples: evidence from the 2013 Demographic and Health Survey. Contracept Reprod Med. 2017;2:9. 10.1186/s40834-017-0037-6. 29201414 10.1186/s40834-017-0037-6 PMC5683226

[31] Adogu P, Udigwe I, Nwabueze A, Adinma E, Udigwe G, Onwasigwe C. Sexual health knowledge, attitude and risk perception among in-school and out-of-school female adolescents in Onitsha, Anambra State, Nigeria. South East Eur J Public Health. 2023 Jan;24. 10.56801/seejph.vi.36.

[32] Adeomi AA, Adeoye OA, Adewole A, Israel OA, Temitayo-Oboh A. Sexual risk behaviors among adolescents attending secondary schools in a Southwestern State in Nigeria. J Behav Health. 10.5455/jbh.20140815092416.

[33] Omisore A, Oyerinde I, Abiodun O, Aderemi Z, Adewusi T, Ajayi I, Fagbolade T, Miskilu S. Factors associated with risky sexual behavior among sexually experienced undergraduates in Osun state, Nigeria. Afr Health Sci. 2022;22(1):41–50. https://www.ajol.info/index.php/ahs/article/view/224553. 36032494 10.4314/ahs.v22i1.6 PMC9382504

[34] Oharume IM. Knowledge, sexual behaviors and risk perception of sexually transmitted infections among students of the polytechnic, Ibadan, Oyo state. Afr Health Sci. 2020;20(1):39–44. https://www.ajol.info/index.php/ahs/article/view/194933. 33402890 10.4314/ahs.v20i1.7 PMC7750043

[35] Adejumo OA, Adebayo BI, Adesola S, Bowale A, Adejumo EN, Atewe S, Sijuade O, Airauhi A, Sodipo O, Shogbamimu Y. Factors associated with risky sexual behavior among clients undertaking HIV testing and counseling services at a secondary referral hospital Lagos. Nigeria Afr Health Sci. 2022;22(1):51–61. https://www.ajol.info/index.php/ahs/article/view/224721. 36032429 10.4314/ahs.v22i1.7 PMC9382506

[36] Eyam LE, Eyam SE, Ekpeyong BN, Ndep AO, Akpan MI, Ekanem EE. Determinants of risky sexual behavior among secondary school adolescents in Cross River State, Nigeria. Niger J Med. 2021;30(6):658–64. https://www.ajol.info/index.php/njm/article/view/220271.

[37] Osuala EO, Udi OA, Ogbu BN, Ojong IN. Risky sexual behavior among undergraduates in Rivers State, Nigeria: A descriptive survey. Afr J Nurs Midwifery. 2021;9(1):001–7. http://www.internationalscholarsjournals.org/.

[38] Okunlola DA, Alawode OA, Bolarinwa OA, Agbeja IO, Awoyele AF. Sociodemographic, economic and psychological correlates of risky sexual behavior among sexually active young people in Nigeria. Global J Health Sci. 10.5539/gjhs.v12n8p9.

[39] Adedini SA, Mobolaji JW, Alabi M, Fatusi AO. Changes in contraceptive and sexual behaviors among unmarried young people in Nigeria: Evidence from nationally representative surveys. PLoS ONE. 2021;16(2):e0246309. 10.1371/journal.pone.0246309. 33529246 10.1371/journal.pone.0246309 PMC7853509

[40] Akokuwebe ME, Falayi EO, Adekola F, Saliu MY. Sexual behavior of in-school rural adolescents in Ogun State. Nigeria Afr J Biomedical Res. 2019;22(2):135–43. https://www.ajol.info/index.php/ajbr/article/view/190603.

[41] Osadolor UE, Amoo EO, Azuh DE, Mfonido-Abasi I, Washington CP, Ugbenu O. Exposure to sex education and its effects on adolescent sexual behavior in Nigeria. J Environmental Public Health. 2022;2022(1):3962011. 10.1155/2022/3962011. 10.1155/2022/3962011 PMC9177302 35692663

[42] Alo OD, Daini BO, Omisile OK, et al. Factors influencing the use of modern contraceptive in Nigeria: a multilevel logistic analysis using linked data from performance monitoring and accountability 2020. BMC Womens Health. 2020;20:191. 10.1186/s12905-020-01059-6. 32883259 10.1186/s12905-020-01059-6 PMC7650288

[43] Chingle MP, Odunze PA, Mohammed A, Bitto TT, Sodipo OY, Zoakah AI. Predictors of male condom utilization in Plateau State, Nigeria. Niger J Clin Pract. 2017;20(9):1079–87. https://www.ajol.info/index.php/njcp/article/view/162412. 29072229 10.4103/njcp.njcp_56_17

[44] Ochonye B, Folayan MO, Fatusi AO, et al. Sexual practices, sexual behavior and HIV risk profile of key populations in Nigeria. BMC Public Health. 2019;19:1210. 10.1186/s12889-019-7553-z. 31477063 10.1186/s12889-019-7553-z PMC6721228

[45] Uchendu OC, Adeyera O, Owoaje ET. Awareness and utilization of female condoms among street youths in Ibadan, an urban setting in South–West Nigeria. Pan Afr Med J. 2019;33(1). https://www.ajol.info/index.php/pamj/article/view/212196. 10.11604/pamj.2019.33.168.12733 PMC6756805 31565129

[46] Odimegwu C, Somefun OD. Ethnicity, gender and risky sexual behavior among Nigerian youth: an alternative explanation. Reprod Health. 2017;14:16. 10.1186/s12978-017-0284-7. 28143542 10.1186/s12978-017-0284-7 PMC5282662

[47] Ajayi AI, Akpan W. Determinants of condom use among parous women in North Central and South Western Nigeria: a cross-sectional survey. BMC Res Notes. 2018;11:467. 10.1186/s13104-018-3573-5. 30005715 10.1186/s13104-018-3573-5 PMC6044001

[48] Bhattacharya D. Public health policy: issues, theories, and advocacy. Hoboken, NJ: Wiley; 2013. p. 21.

[49] Ahmmed F, Chowdhury MS, Helal SM. Sexual and reproductive health experiences of adolescent girls and women in marginalized communities in Bangladesh. Cult Health Sex. 2022;24(8):1035–48. 10.1080/13691058.2021.1909749. 33941046 10.1080/13691058.2021.1909749

[50] Akila D, Oluwasegun A, Bose K, Omotoso O, Adefila A, Mwaikambo L. Improving the Quality of Adolescent and Youth-Friendly Health Services Through Integrated Supportive Supervision in Four Nigerian States. Glob Health Sci Pract. 2024;12(Supplement 2). 10.9745/GHSP-D-22-00169. 10.9745/GHSP-D-22-00169 PMC11111107 38621816

[51] Akuiyibo S, Anyanti J, Idogho O, et al. Impact of peer education on sexual health knowledge among adolescents and young persons in two North Western states of Nigeria. Reprod Health. 2021;18:204. 10.1186/s12978-021-01251-3. 34641895 10.1186/s12978-021-01251-3 PMC8513198

[52] Akande OW, Muzigaba M, Igumbor EU, et al. The effectiveness of an m-Health intervention on the sexual and reproductive health of in-school adolescents: a cluster randomized controlled trial in Nigeria. Reprod Health. 2024;21:6. 10.1186/s12978-023-01735-4. 38218840 10.1186/s12978-023-01735-4 PMC10788027

[53] Usonwu I, Ahmad R, Curtis-Tyler K. Parent–adolescent communication on adolescent sexual and reproductive health in sub-Saharan Africa: a qualitative review and thematic synthesis. Reprod Health. 2021;18:202. 10.1186/s12978-021-01246-0. 34629082 10.1186/s12978-021-01246-0 PMC8504018

[54] Taiwo MO, Oyekenu O, Hussaini R. Understanding how social norms influence access to and utilization of adolescent sexual and reproductive health services in Northern Nigeria. Front Sociol. 2023;8. 10.3389/fsoc.2023.865499. 10.3389/fsoc.2023.865499 PMC10603227 37899781

[55] Meyer D, Heiden-Rootes K, Sledge R, Salas J. The role of couple dynamics in contraception decision-making. Couple Fam Psychol Res Pract. 2022;11(3):120–35. 10.1037/cfp0000226.

[56] Seth K, Nanda S, Sahay A, Verma R, Achyut P. Men, The Missing Link In Gender-equitable Family Planning: A Scoping Review. Gates Open Res. 2022;6(73):73. 10.21203/rs.3.rs-362090/v1.

[57] Aventin Á, Robinson M, Hanratty J, Keenan C, Hamilton J, McAteer ER, et al. Involving men and boys in family planning: A systematic review of the effective components and characteristics of complex interventions in low-and middle‐income countries. Campbell Syst Rev. 2023;19(1). 10.1002/cl2.1296. 10.1002/cl2.1296 PMC9837728 36911859

[58] Vizheh M, Muhidin S, Moghadam B. Women empowerment in reproductive health: a systematic review of measurement properties. BMC Womens Health. 2021;21:424. 10.1186/s12905-021-01566-0. 34930243 10.1186/s12905-021-01566-0 PMC8690621

[59] Abiodun O, Sodeinde K, Jagun O, Ladele A, Adepoju A, Ohiaogu F, et al. Influence of perception of family support and functioning on adolescent high-risk sexual behavior. Am J Trop Med Hyg. 2021;104(3):1153–8. 10.4269/ajtmh.20-0732. 10.4269/ajtmh.20-0732 PMC7941804 33289467

[60] Widman L, Javidi H, Maheux AJ, et al. Sexual Communication in the Digital Age: Adolescent Sexual Communication with Parents and Friends About Sexting, Pornography, and Starting Relationships Online. Sex Cult. 2021;25:2092–109. 10.1007/s12119-021-09866-1.

[61] Maina BW, Ushie BA, Kabiru CW. Parent–child sexual and reproductive health communication among very young adolescents in Korogocho informal settlement in Nairobi, Kenya. Reprod Health. 2020;17:79. 10.1186/s12978-020-00938-3. 32487239 10.1186/s12978-020-00938-3 PMC7268390

[62] Livingston JA, Allen KP, Nickerson AB, et al. Parental Perspectives on Sexual Abuse Prevention: Barriers and Challenges. J Child Fam Stud. 2020;29:3317–34. 10.1007/s10826-020-01796-0.

[63] Ragavan MI, Barral RL, Randell KA. Addressing adolescent relationship abuse in the context of reproductive health care. Semin Reprod Med. 2022;40(01/02):146–54. 10.1055/s-0041-1741519. 34996120 10.1055/s-0041-1741519 PMC9885502

[64] Kaestle CE, Allen KR, Wesche R, Grafsky EL. Adolescent sexual development: A family perspective. J Sex Res. 2021;58(7):874–90. 10.1080/00224499.2021.1924605. 34003063 10.1080/00224499.2021.1924605

[65] Allsop Y, Anderman EM. Developing Sexual Self-Efficacy Beliefs During Adolescence: Do Health Teachers truly Matter? J Youth Adolescence. 2022;51:2061–76. 10.1007/s10964-022-01646-w. 10.1007/s10964-022-01646-w 35794443

[66] Ceylan D, Akan-Celen FN, Özkan S, Aycan Z. Promoting adolescent health: health literacy, self-efficacy and internet use. Turk J Pediatr. 2022;64(1):110–21. 10.24953/turkjped.2021.1264. 35286037 10.24953/turkjped.2021.1264

[67] Voyiatzaki C, Venetikou MS, Papageorgiou E, Anthouli-Anagnostopoulou F, Simitzis P, Chaniotis DI, Adamopoulou M. Awareness, knowledge and risky behaviors of sexually transmitted diseases among young people in Greece. Int J Environ Res Public Health. 2021;18(19):10022. 10.3390/ijerph181910022. 34639324 10.3390/ijerph181910022 PMC8508576

[68] Agimas MC, Solomon M, Shewaye DA, Abebaw Angaw D, Derseh NM. Prevalence of delayed treatment for sexually transmitted infections and its determinants in sub-Saharan Africa. A systematic review and meta-analysis. PLoS ONE. 2024;19(3). 10.1371/journal.pone.0299629. 10.1371/journal.pone.0299629 PMC10956779 38512837

[69] Hsu KK, Rakhmanina NY. Adolescents and young adults: the pediatrician’s role in HIV testing and preand postexposure HIV prophylaxis. Pediatrics. 2022;149(1). 10.1542/peds.2021-055207. 10.1542/peds.2021-055207 PMC9645702 34972226

[70] Huber-Krum S, Karandikar S, Gezinski L. A condom is compulsory: A qualitative study of condom use and negotiation strategies among female sex workers in Nepal. Women Health. 2020;60(8):872–86. 10.1080/03630242.2020.1766641. 32744189 10.1080/03630242.2020.1766641

[71] Saldanha N. STIs in adolescents: Chlamydia, gonorrhea, mycoplasma genitalium, and HPV. Curr Probl Pediatr Adolesc Health Care. 2020;50(7):100835. 10.1016/j.cppeds.2020.100835. 32768342 10.1016/j.cppeds.2020.100835

[72] Escobar DFSS, Noll PRES, Jesus TF, Noll M. Assessing the mental health of Brazilian students involved in risky behaviors. Int J Environ Res Public Health. 2020;17(10):3647. 10.3390/ijerph17103647. 32455911 10.3390/ijerph17103647 PMC7277166

[73] Longo LM, Ertl MM, Pazienza R, Agiliga AU, Dillon FR, Martin JL. Associations among negative urgency, sensation seeking, alcohol use, self-esteem, and casual sexual behavior for college students. Subst Use Misuse. 2020;55(5):796–805. 10.3390/ijerph17103647. 31876218 10.1080/10826084.2019.1703748

[74] Hegbe KG, Réveillère C, Barrault S. Sexual addiction and associated factors: the role of emotion dysregulation, impulsivity, anxiety and depression. J Sex Marital Ther. 2021;47(8):785–803. 10.1080/0092623X.2021.1952361. 34338617 10.1080/0092623X.2021.1952361

[75] Lew-Starowicz M, Lewczuk K, Nowakowska I, Kraus S, Gola M. Compulsive sexual behavior and dysregulation of emotion. Sex Med Rev. 2020;8(2):191–205. 10.1016/j.sxmr.2019.10.003. 31813820 10.1016/j.sxmr.2019.10.003

[76] Claussen C, Exner-Cortens D, Baker E, Roy M, Coupland K. Promotion of sexual health self-efficacy through gender-transformative intervention with adolescent boys. Am J Sex Educ. 2024;19(2):140–66. 10.1080/15546128.2023.2213453.

[77] Rees SN, Crowe M, Harris S. The lesbian, gay, bisexual and transgender communities’ mental health care needs and experiences of mental health services: An integrative review of qualitative studies. J Psychiatr Ment Health Nurs. 2021;28(4):578–89. 10.1111/jpm.12720. 33295065 10.1111/jpm.12720

[78] McGorry PD, Mei C, Chanen A, Hodges C, Alvarez-Jimenez M, Killackey E. Designing and scaling up integrated youth mental health care. World Psychiatry. 2022;21(1):61–76. 10.1002/wps.20938. 35015367 10.1002/wps.20938 PMC8751571

