# Supplementary material for: Risk factors associated with sexually transmitted infections in Nigeria: a systematic review
Source: BMC Public Health. 2026 Mar 9;26:1224. doi: 10.1186/s12889-026-26918-z (PMC13081596; doi:10.1186/s12889-026-26918-z)
Supplement: Supplementary file 1 — Supplementary Material 1. [file 12889_2026_26918_MOESM1_ESM.docx]

**Supplementary Materials**

**Table** 3 Search protocol for various databases

| Search Protocol Component | Details |
| --- | --- |
| Database | PubMed |
| Search Query Steps |  |
| 1 | Adolescent OR youth OR "young adult*" OR adult* OR student* OR "sexually active" OR "young people |
| 2 | exp Sexual Behavior/ OR exp Sexual Health/ OR exp Risk Factors/ OR exp Sexually Transmitted Diseases/ |
| 3 | “STI risk factors” OR “risky sexual behavior” OR “sexual health” OR “sexually transmitted infections” OR sexual behavior OR sexual activity OR sexual practices OR sexual intercourse OR sexual relationships OR sexual risk-taking OR sexual attitudes |
| 4 | 2 OR 3 |
| 5 | “condom use” OR “contraceptive” OR condom usage OR safe sex practices OR birth control OR family planning OR contraception methods OR preventive measures OR reproductive health OR unprotected sex OR sexual protection |
| 6 | Nigeria |
| 7 | 1 AND 4 AND 5 AND 6 |
| Population Keywords | Adolescents, young adults, youths, sexually active, teenagers, youth, juveniles, school-aged, students, young people, emerging adults |
| Topic Keywords | STI risk factors, risky sexual behavior, sexual health, sexually transmitted infections, sexual behavior, sexual activity, sexual practices, sexual intercourse, sexual relationships, sexual risk-taking, sexual attitudes |
| Behavior Keywords | Condom use, contraceptive, condom usage, safe sex practices, birth control, family planning, contraception methods, preventive measures, reproductive health, unprotected sex, sexual protection |
| Location Keywords | Nigeria |
| Boolean Operators | AND, OR |
| Filters Applied | - Publication date: Last ten years<br>- Articles in English<br>- Peer-reviewed articles |
| Inclusion Criteria | Studies focusing on adolescents and young adults in Nigeria, addressing STI risk factors, sexual behaviors, and health practices |
| Exclusion Criteria | Articles not in English, studies focusing on populations outside Nigeria, publications older than ten years |
| Expected Output | Comprehensive list of relevant peer-reviewed articles, reviews, and studies addressing the research question |

**Table** 3 Search protocol for various databases (Continued)

| Search Protocol Component | Details |
| --- | --- |
| Database | Embase |
| Search Query Steps |  |
| 1 | adolescent OR youth OR "young adult*" OR adult* OR student* OR "sexually active" OR "young people" |
| 2 | “STI risk factors” OR “risky sexual behavior” OR “sexual health” OR “sexually transmitted infections” OR "sexual behavior" OR "sexual activity" OR "sexual practices" OR "sexual intercourse" OR "sexual relationships" OR "sexual risk-taking" OR "sexual attitudes" |
| 3 | “condom use” OR “contraceptive” OR "condom usage" OR "safe sex practices" OR "birth control" OR "family planning" OR "contraception methods" OR "preventive measures" OR "reproductive health" OR "unprotected sex" OR "sexual protection" |
| 4 | Nigeria |
| 5 | 1 AND 2 AND 3 AND 4 |
| Population Keywords | Adolescents, young adults, youths, sexually active, teenagers, youth, juveniles, school-aged, students, young people, emerging adults |
| Topic Keywords | STI risk factors, risky sexual behavior, sexual health, sexually transmitted infections, sexual behavior, sexual activity, sexual practices, sexual intercourse, sexual relationships, sexual risk-taking, sexual attitudes |
| Behavior Keywords | Condom use, contraceptive, condom usage, safe sex practices, birth control, family planning, contraception methods, preventive measures, reproductive health, unprotected sex, sexual protection |
| Location Keywords | Nigeria |
| Boolean Operators | AND, OR |
| Filters Applied | - Publication date: Last ten years<br>- Articles in English<br>- Peer-reviewed articles |
| Inclusion Criteria | Studies focusing on adolescents and young adults in Nigeria, addressing STI risk factors, sexual behaviors, and health practices |
| Exclusion Criteria | Articles not in English, studies focusing on populations outside Nigeria, publications older than ten years |
| Expected Output | Comprehensive list of relevant peer-reviewed articles, reviews, and studies addressing the research question |

**Table** 3 Search protocol for various databases (Continued)

| Search Protocol Component | Details |
| --- | --- |
| Database | African Journals Online (AJOL) |
| Search Query Steps |  |
| 1 | adolescent OR youth OR "young adult*" OR adult* OR student* OR "sexually active" OR "young people" |
| 2 | “STI risk factors” OR “risky sexual behavior” OR “sexual health” OR “sexually transmitted infections” OR "sexual behavior" OR "sexual activity" OR "sexual practices" OR "sexual intercourse" OR "sexual relationships" OR "sexual risk-taking" OR "sexual attitudes" |
| 3 | “condom use” OR “contraceptive” OR "condom usage" OR "safe sex practices" OR "birth control" OR "family planning" OR "contraception methods" OR "preventive measures" OR "reproductive health" OR "unprotected sex" OR "sexual protection" |
| 4 | Nigeria |
| 5 | 1 AND 2 AND 3 AND 4 |
| Population Keywords | Adolescents, young adults, youths, sexually active, teenagers, youth, juveniles, school-aged, students, young people, emerging adults |
| Topic Keywords | STI risk factors, risky sexual behavior, sexual health, sexually transmitted infections, sexual behavior, sexual activity, sexual practices, sexual intercourse, sexual relationships, sexual risk-taking, sexual attitudes |
| Behavior Keywords | Condom use, contraceptive, condom usage, safe sex practices, birth control, family planning, contraception methods, preventive measures, reproductive health, unprotected sex, sexual protection |
| Location Keywords | Nigeria |
| Boolean Operators | AND, OR |
| Filters Applied | - Publication date: Last ten years<br>- Articles in English<br>- Peer-reviewed articles |
| Inclusion Criteria | Studies focusing on adolescents and young adults in Nigeria, addressing STI risk factors, sexual behaviors, and health practices |
| Exclusion Criteria | Articles not in English, studies focusing on populations outside Nigeria, publications older than ten years |
| Expected Output | Comprehensive list of relevant peer-reviewed articles, reviews, and studies addressing the research question |

**Table** 3 Search protocol for various databases (Continued)

| Search Protocol Component | Details |
| --- | --- |
| Database | Web of Science |
| Search Query Steps |  |
| 1 | TS=( adolescent OR youth OR "young adult*" OR adult* OR student* OR "sexually active" OR "young people") |
| 2 | TS=("STI risk factors" OR "risky sexual behavior" OR "sexual health" OR "sexually transmitted infections" OR "sexual behavior" OR "sexual activity" OR "sexual practices" OR "sexual intercourse" OR "sexual relationships" OR "sexual risk-taking" OR "sexual attitudes") |
| 3 | TS=("condom use" OR "contraceptive" OR "condom usage" OR "safe sex practices" OR "birth control" OR "family planning" OR "contraception methods" OR "preventive measures" OR "reproductive health" OR "unprotected sex" OR "sexual protection") |
| 4 | TS=Nigeria |
| 5 | #1 AND #2 AND #3 AND #4 |
| Population Keywords | Adolescents, young adults, youths, sexually active, teenagers, youth, juveniles, school-aged, students, young people, emerging adults |
| Topic Keywords | STI risk factors, risky sexual behavior, sexual health, sexually transmitted infections, sexual behavior, sexual activity, sexual practices, sexual intercourse, sexual relationships, sexual risk-taking, sexual attitudes |
| Behavior Keywords | Condom use, contraceptive, condom usage, safe sex practices, birth control, family planning, contraception methods, preventive measures, reproductive health, unprotected sex, sexual protection |
| Location Keywords | Nigeria |
| Boolean Operators | AND, OR |
| Filters Applied | - Publication date: Last ten years<br>- Articles in English<br>- Peer-reviewed articles |
| Inclusion Criteria | Studies focusing on adolescents and young adults in Nigeria, addressing STI risk factors, sexual behaviors, and health practices |
| Exclusion Criteria | Articles not in English, studies focusing on populations outside Nigeria, publications older than ten years |
| Expected Output | Comprehensive list of relevant peer-reviewed articles, reviews, and studies addressing the research question |

The search protocol commenced with PubMed, leveraging its advanced search interface and comprehensive database of biomedical literature. The search query was meticulously constructed, utilizing Boolean operators to combine search terms effectively. Filters were applied to restrict results to recent publications (within the last ten years) and to peer-reviewed articles published in English, ensuring the retrieval of high-quality, relevant literature.

Subsequently, the protocol was adapted for Embase, a prominent biomedical and pharmacological database known for its extensive coverage of scholarly articles. Similar to PubMed, the search query was carefully crafted, employing Boolean operators to combine search terms effectively. Filters were applied to limit results to recent publications and peer-reviewed articles in English. Embase's advanced search functionalities, such as medical subject headings (MeSH) and additional filters for study types, were utilized to refine the search and retrieve relevant literature within the biomedical and pharmacological context.

For African Journals Online (AJOL), similar to other academic databases, advanced search functionalities were utilized. Filters were applied to narrow down results to publications from the last ten years and peer-reviewed articles in English. This ensured the retrieval of relevant literature while maintaining quality standards.

Lastly, the protocol was tailored for Web of Science, a premier multidisciplinary citation database. Utilizing the "TS" field tag, which searches within the title, abstract, author keywords, and Keywords Plus®, ensured comprehensive coverage. Proper use of Boolean operators and application of available filters further refined search results, focusing on recent publications and peer-reviewed articles in English.

**JBI** **Critical Appraisal Checklist for Qualitative Research**

1.Is there congruity between the stated philosophical perspective and the research methodology?

2.Is there congruity between the research methodology and the research question or objectives?

3.Is there congruity between the research methodology and the methods used to collect data?

4.Is there congruity between the research methodology and the representation and analysis of data?

5.Is there congruity between the research methodology and the interpretation of results?

6.Is there a statement locating the researcher culturally or theoretically?

7.Is the influence of the researcher on the research, and vice- versa, addressed?

8.Are participants, and their voices, adequately represented?

9.Is the research ethical according to current criteria or, for recent studies, and is there evidence of ethical approval by an appropriate body?

10.Do the conclusions drawn in the research report flow from the analysis, or interpretation, of the data?

**Table** 4. Quality of Qualitative Research Literatures

| Author | Appraisal questions | | | | | | | | | | | Remark |
| --- | --- | --- | --- | --- | --- | --- | --- | --- | --- | --- | --- | --- |
|  | **1** | **2** | **3** | **4** | **5** | **6** | **7** | **8** | **9** | **10** | **Score** |  |
| Folayan | Yes | Yes | Yes | Yes | Yes | Unclear | Unclear | Yes | Yes | Yes | 7 | Moderate |
| Mbachu *et al.* | Yes | Yes | Yes | Yes | Yes | Unclear | Unclear | Yes | Yes | Yes | 7 | Moderate |

**JBI Critical Appraisal Checklist for Analytical Cross-Sectional Studies**

1.Were the criteria for inclusion in the sample clearly defined?

2.Were the study subjects and the setting described in detail?

3.Was the exposure measured in a valid and reliable way?

4.Were objective, standard criteria used for measurement of the condition?

5.Were confounding factors identified?

6.Were strategies to deal with confounding factors stated?

7.Were the outcomes measured in a valid and reliable way?

8.Was appropriate statistical analysis used?

**Table** 5. Quality of Qualitative Research Literatures

| Author | Appraisal questions | | | | | | | | | Remark |
| --- | --- | --- | --- | --- | --- | --- | --- | --- | --- | --- |
|  | **1** | **2** | **3** | **4** | **5** | **6** | **7** | **8** | **Score** |  |
| Akamike *et al* | Yes | Yes | Yes | Yes | Yes | Yes | Yes | Yes | 8 | High |
| Ajayi *et al.* | Yes | Yes | Yes | Yes | Yes | Yes | Yes | Yes | 8 | High |
| Ajayi and Okeke | Yes | Yes | Yes | Yes | Yes | Yes | Yes | Yes | 8 | High |
| Blackstone & Iwelunmor | Yes | Yes | Yes | Yes | Yes | Yes | Yes | Yes | 8 | High |
| Adogu *et al.* | Yes | Yes | Yes | Yes | Yes | No | Yes | Yes | 7 | High |
| Adeomi *et al.* | Yes | Yes | Yes | Yes | Unclear | No | Yes | Yes | 6 | Moderate |
| Omisore *et al.* | Yes | Yes | Yes | Yes | Yes | Yes | Yes | Yes | 8 | High |
| Oharume | Yes | Yes | Yes | Yes | No | No | Yes | Yes | 6 | Moderate |
| Adejumo *et al.* | Yes | Yes | Yes | Yes | Yes | Yes | Yes | Yes | 8 | High |
| Eyam *et al.* | Yes | Yes | Yes | Yes | Yes | Yes | Yes | Yes | 8 | High |
| Osuala *et al.* | Yes | Yes | Yes | Unclear | No | No | Yes | Yes | 5 | Moderate |
| Okunlola *et al.* | Yes | Yes | Yes | Yes | Yes | Unclear | Yes | Yes | 7 | High |
| Adedini *et al.* | Yes | Yes | Yes | Yes | Yes | Yes | Yes | Yes | 8 | High |
| Akokuwebe *et al.* | Yes | Yes | Yes | Yes | Yes | No | Yes | Yes | 7 | High |
| Osadolor *et al.* | Yes | Yes | Yes | Yes | No | Unclear | Yes | Yes | 6 | Moderate |
| Alo *et al.* | Yes | Yes | Yes | Yes | Yes | Yes | Yes | Yes | 8 | High |
| Chingle *et al.* | Yes | Yes | Yes | Yes | Yes | Yes | Yes | Yes | 8 | High |
| Ochonye *et al.* | Yes | Yes | Yes | Yes | Yes | No | Yes | Yes | 7 | High |
| Uchendu *et al.* | Yes | Yes | Yes | Yes | Yes | No | Yes | Yes | 7 | High |
| Odimegwu & Somefun | Yes | Yes | Yes | Yes | Yes | Yes | Yes | Yes | 8 | High |
| Ajayi and Akpan | Yes | Yes | Yes | Yes | Yes | Yes | Yes | Yes | 8 | High |
